# Protocol for a feasibility study and randomised pilot trial of a low-intensity psychological intervention for depression in adults with autism: the Autism Depression Trial (ADEPT)

**DOI:** 10.1136/bmjopen-2017-019545

**Published:** 2017-12-03

**Authors:** Ailsa Russell, Kate Cooper, Stephen Barton, Ian Ensum, Daisy Gaunt, Jeremy Horwood, Barry Ingham, David Kessler, Chris Metcalfe, Jeremy Parr, Dheeraj Rai, Nicola Wiles

**Affiliations:** 1 Department of Psychology, University of Bath, Bath, UK; 2 Newcastle Cognitive and Behaviour Therapies (CBT) Centre, Northumberland Tyne & Wear NHS Foundation Trust, Newcastle upon Tyne, UK; 3 School of Psychology, Newcastle University, Newcastle upon Tyne, United Kingtom; 4 Bristol Adult Autism Services, Avon and Wiltshire Mental Health Partnership NHS Trust, Chippenham, UK; 5 Population Health Sciences, Bristol Randomised Trials Collaboration, Bristol Medical School, University of Bristol, Bristol, UK; 6 Northumberland, Tyne & Wear NHS Foundation Trust, Northumberland, UK; 7 Institute of Neuroscience, University of Newcastle, Newcastle, UK; 8 Population Health Sciences, School of Social and Community Medicine, Bristol Medical School, University of Bristol, Bristol, UK; 9 Centre for Academic Mental Health, Population Health Sciences, Bristol Medical School, University of Bristol, Bristol, UK

## Abstract

**Introduction:**

High rates of co-occurring depression are reported in autism spectrum disorder (ASD), a neurodevelopmental condition characterised by social communication impairments and repetitive behaviours. Cognitive-behavioural interventions adapted for ASD have been effective for anxiety problems. There have been evaluation studies of group cognitive-behavioural therapy for co-occurring depression, but no randomised trials investigating low-intensity psychological interventions as recommended in clinical guidelines for mild-moderate depression.

**Methods and analysis:**

A feasibility study comprising a randomised controlled trial (RCT) and nested qualitative evaluation is under way as preparation for a definitive RCT. Participants (n=70) will be randomised to Guided Self-Help: a low-intensity psychological intervention based on behavioural activation adapted for ASD or treatment as usual. Outcomes including depression symptoms, anxiety, social function and service use will be measured at 10, 16 and 24 weeks postrandomisation and will be blind to group allocation for measures that are not self-administered. The analysis will aim to establish the rates of recruitment and retention for a larger-scale RCT as well as the most appropriate measure of depression to serve as primary outcome. The qualitative study will purposively sample up to 24 participants from each treatment group to consider the acceptability and feasibility of the intervention and the trial design.

**Ethics and dissemination:**

Ethical approval has been received from WALES REC 3 (IRAS project ID: 191558) and the Health Research Authority with R&D approval from Avon and Wiltshire Mental Health Partnership and Northumberland, Tyne and Wear Foundation NHS Trusts. To our knowledge, this is the first study of a low-intensity intervention for depression in adults with autism. The results will inform the design of a definitive RCT. Dissemination will include peer-reviewed journal publications reporting the quantitative and qualitative research findings of the study and presentations at national and international conferences.

**Trial registration number:**

ISRCTN54650760; Pre-results.

**Strengths and limitations of this study**

- This trial is funded following a commissioned call and thus aims to fill a clearly perceived clinical research and service gap.
- Recruitment procedures ensure diagnostic validity of autism spectrum disorder (ASD) and depression.
- Conclusions about effectiveness or efficacy of the intervention are not possible.
- Inclusion of clinician as well as self-report measures of depression symptoms aims to overcome the documented difficulties with self-report of emotional states in ASD.
- Patient and public involvement in the development of the intervention.

## Background

Autism spectrum disorder (ASD) is a neurodevelopmental condition characterised by qualitative impairments in social communication and a pattern of restricted, repetitive behaviour, interests or activities.^1^ High rates of co-occurring mental health problems have been reported in studies of children^2^ and adults^3^ with ASD. These include depression, a common mental disorder characterised by persistent low mood, feelings of guilt and low self-worth, an absence of positive affect and disturbances in sleep, appetite, concentration and energy levels. Depression is a debilitating mental health problem and has been estimated as the leading cause of loss of disability-adjusted life years due to mental health and substance use disorders.^4^ The estimated point prevalence for a depressive episode for adults aged 16–74 in the UK general population in 2000 was 2.6%.^5^ To date, there have been no robust epidemiological studies of depression in ASD, but studies of clinical and research samples have reported high rates. For example, a clinical study of 122 autistic adults with ASD found that 53% met criteria for depression at some point in their lives.^6^ A recent systematic review reported rates of depression ranging from 4% to 35% across seven studies of autistic adults without intellectual disability.^7^ Although sampling and measurement methods may contribute to the lack of consistent findings across studies, there is some evidence to suggest that the prevalence of depression is notably higher in autistic adults than the general population.

The National Institute for Health and Care Excellence (NICE) Clinical Guidelines 90^8^ recommend a stepped-care model for the treatment of depression. Mild-moderate depression should be treated according to step 2 of the care pathway, that is, low-intensity psychosocial interventions, psychological interventions, medication and referral for further assessment and treatment. Low-intensity interventions aim to increase access to evidence-based psychological therapies. They are characterised by the provision of evidence-based information, delivered by a range of ‘technologies’ such as written materials or computerised protocols, and facilitated by a mental health worker without formal professional training or self-facilitated. Monitoring and review are also important. Low-intensity psychological interventions for depression as recommended by the clinical guidelines^8^ are individual guided self-help based on the principles of cognitive-behavioural therapy (CBT) to include behavioural activation and problem-solving techniques, computerised CBT or a structured group physical activity programme.

CBT refers to a group of psychological interventions which combine behavioural, cognitive and affect-focused techniques to bring about a reduction in psychological distress and an improvement in associated functional impairment. Adaptations to the delivery and content of CBT are outlined in the clinical guidance for adults with autism.^9^ Recent systematic reviews indicate that CBT can be effective in reducing anxiety problems in young people^10 11^ and adults with ASD^12^ if adapted. Meta-analysis of the studies of adapted CBT interventions for emotional disorders in children and adults report small–medium effect sizes (g*=*0.24 on self-report outcomes, g*=*0.66 on informant measures and g*=*0.73 on clinician rated outcomes).^13^

Despite the high rates of co-occurring depression and the success in evaluating the effectiveness of adapting CBT for anxiety problems, there is a paucity of systematic evidence for its usefulness in treating depression in ASD. There have been two randomised evaluations of group CBT for young people with ASD using wait-list and cross-over trial designs^14 15^ with mixed findings reported in respect of changes on the primary outcome measures and relatively small sample sizes. An adult study^16^ reported a significant effect of time but not treatment group on anxiety and depression measures when comparing a CBT intervention with mindfulness-based stress reduction both adapted for ASD. There are then suggestions that CBT adapted for ASD may be effective for adults with co-occurring depression, but to our knowledge, there have been no published clinical effectiveness studies of individual cognitive-behavioural interventions, including behavioural activation or of a low-intensity intervention.

Clinical trials aside, naturalistic treatment evaluations are less well described and reported. Thus, it is not known whether adults with ASD and co-occurring mild-moderate depression routinely access CBT interventions offered in primary care or experience less favourable treatment outcomes. It is known that the materials for low-intensity CBT for depression have not been specifically developed with this group in mind.

### Aims

As part of a commissioned call by the National Institute for Health Research Health Technology Assessment, this was a feasibility study for a large-scale randomised controlled trial (RCT) that would determine the clinical and cost-effectiveness of a low-intensity intervention for co-occurring depression in adults with autism. The aims of the feasibility study were to:

- Develop a low-intensity intervention for depression adapted for ASD based on NICE recommendations and accompanying training materials to guide therapists in supporting the intervention.
- Investigate the feasibility and patient and therapist acceptability of the low-intensity intervention.
- Estimate the rates of recruitment and retention for a large-scale RCT.
- Identify the most appropriate outcome measure for a large-scale RCT.

## Methods

### Trial design

The trial will comprise a single-blind, RCT, with a nested qualitative evaluation. Participants will be randomly assigned to one of two treatment arms, a low-intensity intervention, Guided Self-Help for depression adapted for adults with autism (GSH), or treatment as usual (TAU).

### Recruitment

Participants (n=70) will be recruited from Adult Autism Clinics (n=2) and research organisations for adults with ASD (n=2).

#### Inclusion criteria

- aged ≥18 years
- clinical diagnosis of ASD
- current depression as measured by Patient Health Questionnaire (PHQ-9) score ≥10.

#### Exclusion criteria

- participants who are non-English speaking
- participants with literacy levels such that the written materials are inaccessible
- risk of suicide such that a low-intensity intervention is not in line with clinical need
- history of psychosis
- current alcohol/substance dependence
- untreated epilepsy
- attended >6 sessions of individual CBT during the previous 6 months.

### Procedure

Clinicians in the NHS Adult Autism Clinics will introduce the study to potentially eligible participants.

Coordinators of the two research pathways will introduce the study to potentially eligible participants by mailshot, inviting people to find out more about the study according to the research opportunity protocols and guidelines.

Potentially eligible participants who express an interest in the study will be invited to contact the research team to find out more. Eligibility screening according to the inclusion and exclusion criteria will then be conducted over telephone. Participants meeting the inclusion/exclusion criteria will be invited to a meeting where eligibility will be confirmed using the Clinical Interview Schedule—Revised (CIS-R),^17^ a standardised interview conducted by the researcher.

Fully informed, written consent to participate in the study will be sought. The researcher will ask participants to summarise in their own words what taking part in the study will involve, including enquiring about their understanding of the voluntary nature of the study.

Baseline assessment will be completed face to face for all consenting participants and include a number of self-report measures of depression, anxiety, obsessive–compulsive symptoms, quality of life, social function, rumination, repetitive behaviours and a current use of services questionnaire (see the Measures section). A clinician-administered depression measure will also be completed, the Hamilton Rating Scale for Depression (HRSD).

A preferred communication and research contact strategy will be agreed with each individual at the outset of their participation in the study. As well as working with individual preferences, the aim is to minimise any retention issues occurring as a result of core characteristics of ASD. Individual preferences may involve carers or other family members, a sole reliance on electronic communication, repeated reminders etc.

### Randomisation

**Figure 1:**
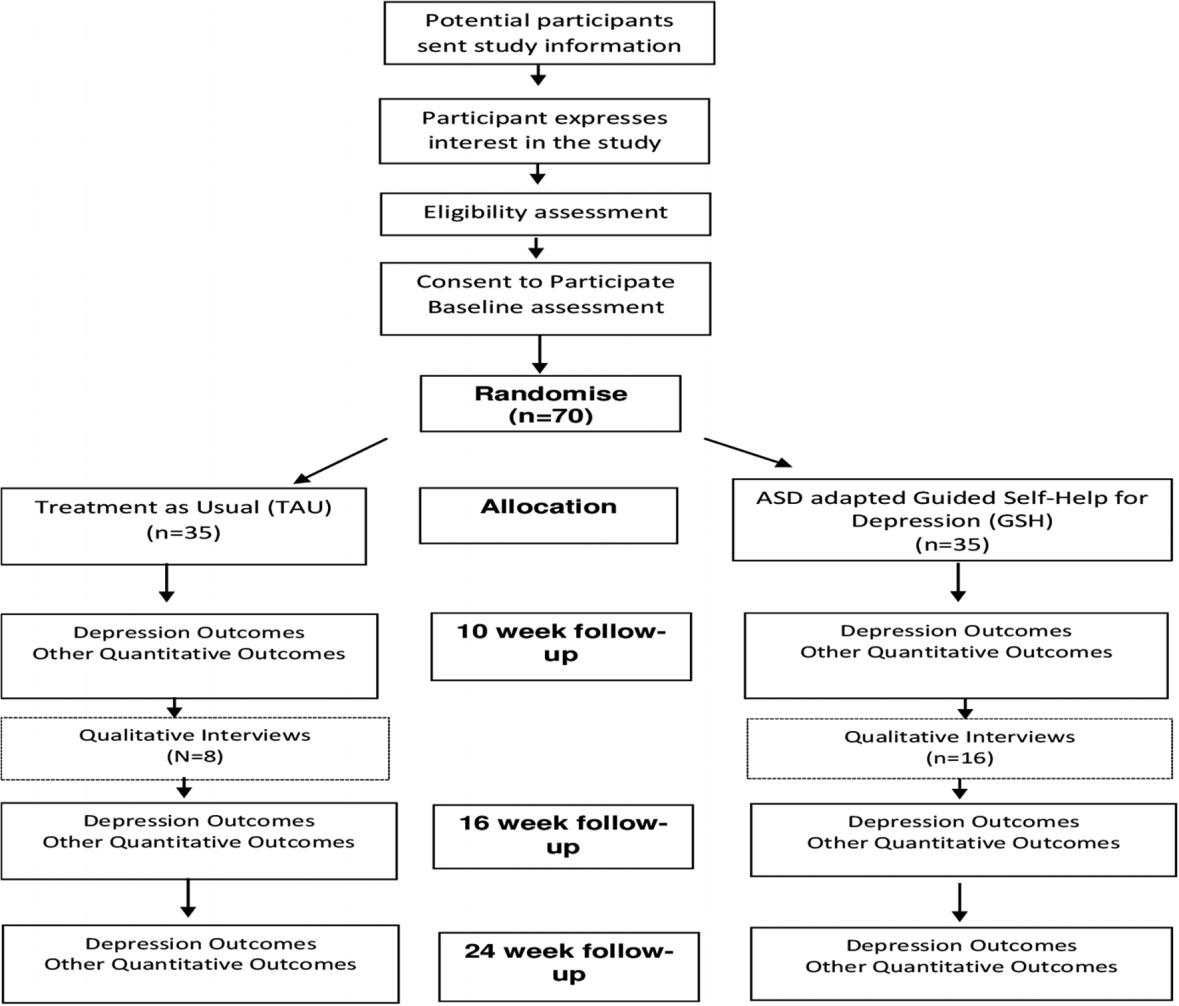
Timeline of events. ASD, autism spectrum disorder; GSH, Guided Self-Help; TAU, treatment as usual.

Eligible and consenting participants will be randomly allocated to GSH (n=35) or TAU (n=35) via a remote computerised randomisation service. Randomisation will be stratified by NHS regional centre (n=2), and minimised by depression severity (mild-moderate, ie, PHQ-9 scores between 10 and 15 or moderate-severe, ie, PHQ-9 scores between 16 and 27) and antidepressant medication (currently taking/not taking). The trial manager will share details of the outcome of randomisation with the individual participants according to their communication preferences within 48 hours and with the therapist. Follow-up measurement will be carried out by researchers who will remain blind to treatment allocation (see figure 1 for timeline of events).

### Randomised treatments

#### Guided Self-Help

The intervention is based on the principles of behavioural activation for depression, adapted for ASD and delivered as a low-intensity intervention.

The intervention will be delivered over 10 weeks. The first session (0) is a planning session to orient the participant to the nature of guided self-help and the therapist to the nature of the participant’s ASD. Individualised goals for treatment are formulated during this session. Sessions 1–4 contain information and tasks to facilitate learning about the importance of links between situations, behaviour and feelings. Activity scheduling to increase opportunities for positive mood, in line with treatment goals, form the basis for sessions 5–8. Session materials are written with visual cues to enhance the accessibility of the text. A consistent session format is maintained throughout the treatment. Feedback and advice about the materials during development was obtained from two adults with ASD. They provided feedback about the clarity and acceptability of the written text, accompanying visual images, suggested homework tasks, the content of examples used to convey concepts, the visual layout and format of the written materials, and the amount of material provided for each individual session. A focus group of adults with ASD was then asked to provide feedback on the final version of the planning session and the study information sheets.

Therapist ‘guides’ will facilitate the sessions, encouraging participants to review and reflect on the session materials, and plan homework tasks. Therapist guides will be unqualified psychologists with a foundation knowledge in CBT consistent with the knowledge and skills of low-intensity therapists. They will include assistant psychologists and clinical psychologists in training. They will not ordinarily have the knowledge and training to deliver individual, formulation-driven CBT. Therapists will receive 15 hours of training in the intervention and 1 hour minimum of clinical supervision on a weekly basis. Supervision will be delivered by qualified clinical psychologists who have developed the intervention. It can be face to face or remote (via telephone/Skype) and can be delivered on an individual or group basis. A therapist manual has been developed for the intervention.

The intervention will be delivered face to face, with sessions lasting up to 45 min. The exception is the initial planning session which can last up to 90 min to facilitate engagement. The intervention can be delivered by telephone or Skype if it is otherwise inaccessible to a participant. However it is designed to be delivered face to face, with the latter sessions (5–8) amenable to delivery by telephone if preferred. Participants who attend ≥5 sessions will be considered to have received a minimally effective dose of treatment.

Sessions will not be audio or video recorded. Therapists will complete a session record at the end of each session listing the treatment components covered in the session, engagement with the session, ease of completion of the session tasks and extent to which any homework tasks were completed. This will allow post hoc evaluation of any logistical and pragmatic issues arising with the session materials. Progress with individual client treatment goals will be rated by the client on a scale of 0–10 in sessions 0, 4 and 8.

Session by session measures will be completed, comprising the PHQ-9, General Anxiety Disorder Questionnaire (GAD-7), brief Positive and Negative Affect Scale and engagement with daily activities. This will simulate routine practice in low-intensity interventions where ongoing monitoring is an important element.

#### Treatment as usual

There will be no restrictions on the care that can be provided as TAU. Participants randomised to TAU will be provided with information about how to self-refer to local Improving Access to Psychological Therapies (IAPT) services. TAU may comprise no treatment, self or general practitioner (GP) referral to IAPT services, Adult Autism Clinic recommending GP referral to IAPT services, Adult Autism Clinic or GP referral to secondary mental health services and/or antidepressant medication. TAU will be carefully recorded in terms of the timing and nature of any intervention received.

### Eligibility measure

*CIS-R* 17: The CIS-R is a structured diagnostic interview which generates International Classification of Disease-10 (ICD-10) psychiatric diagnoses. The CIS-R is a widely used, well-validated diagnostic instrument. The CIS-R was administered via a computerised questionnaire in this study to assess the inclusion and exclusion criteria alongside the PHQ-9 score.

### Outcome measurement

Follow-up assessment will be conducted at 10, 16 and 24 weeks postrandomisation (see table 1 for a full schedule of assessment measures by time point) and will be carried out by researchers blind to treatment allocation. One aim of the study is to identify the most appropriate outcome measure for a large-scale RCT, thus, it is not possible to specify the primary outcome measure of depression a priori. The feasibility of delivering the intervention over 10 weeks will be evaluated. Thus it is not clear if 10 or 16 weeks postrandomisation will represent the most effective point of outcome measurement, that is, if the majority of participants are unable to complete the intervention within 10 weeks.

**Table 1:** Timing of outcome measurement

Measure	Baseline	GSH group each session	10 weeks (end of intervention)	16 weeks	24 weeks
Demographics	√				
PHQ-9	√	√	√	√	√
CIS-R	√				
BDI	√		√	√	√
GRID HAM-D	√		√	√	√
GAD-7	√	√	√	√	√
OCI-R	√		√	√	√
PANAS	√	√	√	√	√
WSAS	√	√	√	√	√
SF-12	√		√	√	√
EQ-5D-5L	√		√	√	√
Participant Global Rating of Change				√	√
RBQ-2A	√				
RRQ	√		√		
Economic evaluation			√	√	√

BDI, Beck Depression Inventory; CIS-R, Clinical Interview Schedule-Revised; EQ-5D-5L, EUROQOL 5 dimensions 5 levels; GAD-7, General Anxiety Disorder Questionnaire; GRID-HAM-D, GRID Hamilton Rating Scale for Depression; GSH, guided self help; OCI-R, Obsessive Compulsive Inventory-Revised; PANAS, Positive and Negative Affect Schedule; PHQ-9, Patient Health Questionnaire; RBQ-2A, Repetitive Behaviours Questionnaire; RRQ, Rumination-Reflection Questionnaire; SF-12, 12-Item Short Form Health Survey; WSAS, Work and Social Adjustment Scale.

### Depression measures

*PHQ-9* 18: The PHQ-9 is a nine-item self-report measure of depression, which is commonly used in primary care settings. The PHQ-9 has been found to be reliable (Cronbach’s α=0.84–0.93), valid and sensitive to change in the general population.^19^ To the authors’ knowledge the psychometric properties of the scale have not been investigated with the autistic population.

*Beck Depression Inventory-II (BDI-II)* 20: The BDI-II is a widely used, 21-item self-report measure of depression. The BDI-II has been found to be reliable (Cronbach’s α=0. 92) for outpatients.^20^ A validation study of 50 young people with ASD^21^ reported good internal consistency on the BDI-II (Cronbach’s α=0.90).

*GRID-Hamilton Rating Scale for Depression (GRID-HAMD-17)* 22: The GRID-HAMD is a version of the HRSD.^23^ It is a 17-item clinician-administered interview which has been found to be reliable and valid in the general population^22^ but to our knowledge has not been investigated in the autistic population. GRID-HAMD interviews will be audio recorded with participant consent. Recordings will be independently rated by a second rater for all items (excluding items 8 and 9 which require face-to-face assessment, ie, observation of psychomotor retardation and agitation) to establish reliability of each assessor. This will be done for the first six interviews conducted by each assessor. To establish reliability across the study, a random sample (20%) of GRID-HAMD recordings will be independently rated.

*Participant Global Rating of Change*: Participants are asked to rate whether their depression is better, worse or much the same on a five-point scale at 16-week and 24-week follow-up.

### Other measures

*GAD-7* 24: The GAD-7 is a seven-item self-report measure of anxiety. The scale has been found to be reliable and valid in the typically developing population^24^ (Cronbach’s α=0.92). However the psychometric properties for use with autistic individuals are not known.

*Positive and Negative Affect Schedule (PANAS)* 25: The PANAS is a 20-item self-report scale of positive and negative affect. The scale has been found to be reliable with a Cronbach’s α of 0.89 for the positive affect scale and 0.85 for the negative affect scale^25^ but has not been used with autistic individuals.

*Obsessive–Compulsive Inventory—Revised (OCI-R)* 26: The OCI-R is an 18-item self-report measure of the symptoms of obsessive–compulsive disorder, with items such as ‘I feel I have to repeat certain numbers’, which are scored on a five-point Likert scale (0–4), indicating increasing frequency. The scale has been found to be reliable and valid.^26^ The OCI-R has been found to have good psychometric properties in a sample of 225 autistic people.^27^

*Work and Social Adjustment Scale (WSAS)* 28: The WSAS is a five-item self-report measure of impaired functioning which has been found to be reliable (Cronbach’s α=0.7–0.94) and valid in typically developing individuals. It has been used with autism populations, but its psychometric properties have not been assessed.

*EQ-5D-5L* 29: The EQ-5D-5L is a five-item self-report measure of health, with items measured on a five-point Likert scale (1–5) indicating severity. It has been found to be reliable and valid in the typically developing population.^30^

*12-Item Short Form Health Survey (SF-12)* 31: The SF-12 is a 12-item self-report measure of physical and mental health. It has been found to be a reliable and valid measure among people with severe mental health problems,^32^ but psychometric properties for autistic populations are not available.

*The Repetitive Behaviours Questionnaire (RBQ-2A)* 33: The RBQ-2A is a 20-item self-report measure of repetitive behaviours. It has been found to have good internal consistency, with Cronbach’s α between 0.73 and 0.83, and autistic people score significantly higher on the measure than typically developing individuals, suggesting that it is a valid measure of autism-specific repetitive behaviours.

*The Rumination–Reflection Questionnaire (RRQ)* 34: The RRQ is a 12-item self-report questionnaire, comprising two subscales; rumination and reflection. The measure has good psychometric properties, and the rumination scale has a Cronbach’s α of 0.90, and the reflection scale 0.91.

*Economic evaluation*: We will pilot the feasibility of data collection on statutory health and voluntary service use using a questionnaire collecting information on: use of other primary and community care services (NHS Direct, attendances at walk-in centres, use of community healthcare services); secondary care related to mental health (number of outpatient visits, type of clinic and reason for visit; inpatient stays, length of stay and reason); use of social services and disability payments received; personal costs related to mental health (expenditure on over-the-counter medication, expenditure on prescriptions, travel costs associated with healthcare visits, loss of earnings, out of pocket expenditure on other services, eg, private counselling or complementary or alternative therapies, child care and domestic help); time off work and unpaid activities. We will also access GP records to provide information on: number of primary care consultations, by type for example, face-to-face, telephone etc, and who seen and prescribed medication.

### Statistical analysis

We will be following the recently published extension to Consolidated Standards of Reporting Trials (CONSORT)^35^ when reporting the results of this feasibility trial. As this is a feasibility study there is no formal sample size calculation. Seventy participants were considered a large enough sample to consider the practicalities of recruitment and delivering the intervention.

For this feasibility study, we will calculate and present in a CONSORT flow chart:

1. the proportion of adults with ASD consenting to the study;
2. the proportion completing the baseline assessment and entering the randomised phase;
3. for those in the intervention group, the number of guided self-help sessions attended and the proportion completing five or more sessions;
4. the proportion completing follow-up assessments at 10, 16 and 24 weeks postrandomisation.

We will explore the sensitivity to change of the various self-reported measures of depression in comparison with the GRID-HAMD (clinician-rated assessment of depression) in order to identify the most appropriate outcome measures for the main trial. We will also compare the continuous scores on the depression outcome measures between groups. The SD of the chosen primary outcome will inform the sample size calculation for the large-scale RCT.

We will evaluate the service use and economical evaluation questionnaire by examining the rates of completion across individual questions. We will compare the information provided by participants about primary and secondary care health service use with information gathered from GP and secondary care records.

### Qualitative study

In-depth interviews will be conducted with participants (from both arms of the trial) 10 weeks after randomisation (after 10 week outcome measurement is complete). These interviews will consider and compare views and experiences of the trial and the acceptability of the guided self-help intervention. All therapists delivering the intervention will also be interviewed towards the end of the trial to illuminate the perceived effectiveness and acceptability of treatments and explore any barriers to its uptake outside of the trial.

Purposive sampling will select interviewees in order to attempt to capture maximum variation in views and experiences in order that they adequately reflect those of a range of participants. All participants in the trial will be asked if they are willing to be contacted about taking part in a qualitative interview at the time of trial consent. From participants who indicate that they are willing to be contacted, a purposive sample will be drawn in relation to (1) the trial site, (2) arm of the trial and (3) sociodemographic variables such as age, gender, ethnicity and socioeconomic status (with participants being selected from areas of high and low social–economic deprivation, based on Index of Multiple Deprivation (IMD 2007) score.^36^ Interviews will be analysed in batches, and sampling will continue until no new themes are emerging from the interviews. This is likely to include up to 24 participants as well as two to three therapist interviews.

All interviews will be conducted by telephone or face-to-face in a location of the participants’ choice. At interview, a flexible topic guide will be used to ensure primary issues are covered during all interviews, but without dictating data collection, allowing participants to introduce unanticipated issues. Interviews are expected to last between 45–60 min. With informed consent, interviews will be recorded using a digital voice recorder, transcribed and anonymised.

Interview transcripts will be checked for accuracy and then imported into NVivo10 qualitative data analysis software,^37^ to aid management and indexing of data. Thematic analysis^38^ using a data-driven inductive approach^39^ will be used to scrutinise the data in order to identify and analyse patterns and themes of particular salience for participants and across the dataset using constant comparison techniques.^40^ Analysis will begin shortly after data collection starts, will be ongoing and iterative. Analysis will inform further data collection; for instance, analytical insights from data gathered in earlier interviews will help identify any changes that need to be made to the topic guide during later interviews. Transcripts from the participants and therapists’ interviews will be analysed separately, with coding frames being developed for each. A subset of transcripts will be independently double-coded by other members of the research team and compared. Discrepancies will be discussed and resolved to achieve a coding consensus.

### Monitoring and adverse events

Adverse events and risk standardised operating procedures have been developed and will be followed by all researchers and therapists working on the trial. Adverse events are defined as significant negative episodes, or significant deterioration in condition, which happen to participants during their time in the trial. These will be reported by research assistants and trial therapists to senior trial staff, who will ascertain whether these are thought to be linked to participation in the trial, and keep records of each event on an adverse events database. All serious adverse events of an unexpected and unrelated nature will be reported to the main Research Ethics Committee, the study Sponsor and Trial Steering Committee (TSC). Suicide risk will be monitored using the suicidality items on the PHQ-9 and BDI-II which are administered at baseline, and 10, 16 and 24-week follow-up. If the participant states that they have had suicidal/self-harming thoughts every day in the last week (PHQ-9) or that they would like to kill themselves, or would kill themselves if they had chance (BDI-II), then a letter will be sent to their GP highlighting the individual’s risk. If the individual is considered to be high risk, a qualified clinician may also follow-up by offering information to the participant regarding local crisis teams, and call the GP to ensure the information is shared in a timely manner.

### Independent oversight

A TSC and Data Monitoring and Ethics Committee will have independent oversight of the study, meeting at 6 monthly intervals with quarterly reports of adverse and serious adverse events.

### Potential limitations

The majority of participants in this study will receive a diagnosis of ASD during adulthood and thus may not be representative of all adults with ASD.

TAU may vary considerably by geographical region and service factors may act as a potential confound.

## Summary

High rates of co-occurring depression, a debilitating mental health problem, have been reported in adults with ASD.^6^ There has been little research to date about the usefulness of adapted CBT for depression in autism or about routine service outcomes for this group. This feasibility study aims to develop a low-intensity intervention for depression for adults with autism based on cognitive-behavioural principles and accompanying therapist guidelines and training. The study design will enable the research team to consider the feasibility of carrying out a large-scale RCT to consider the effectiveness of the intervention, including specifying the most appropriate primary outcome measure. The nested qualitative study will address issues of patient and therapist acceptability of the intervention.

### Trial status

The pilot RCT is currently open to recruitment and the last participant is scheduled to be randomised by the end of September 2017. The trial will run until the end of April 2018.

The full trial protocol can be accessed at NIHR Journals Library (https://www.journalslibrary.nihr.ac.uk/programmes/hta/144302#/).

## Supplementary Material

Reviewer comments

Author's manuscript
