# Supplementary material for: Protocol for a feasibility study and randomised pilot trial of a low-intensity psychological intervention for depression in adults with autism: the Autism Depression Trial (ADEPT)
Source: BMJ Open. 2017 Dec 3;7(12):e019545. doi: 10.1136/bmjopen-2017-019545 (PMC5736092; doi:10.1136/bmjopen-2017-019545)
Supplement: Author's manuscript [file bmjopen-2017-019545.draft_revisions.pdf]

BMJ Open is committed to open peer review. As part of this commitment we make the peer review history of every article we publish publicly available.

When an article is published we post the peer reviewers' comments and the authors' responses online. We also post the versions of the paper that were used during peer review. These are the versions that the peer review comments apply to.

The versions of the paper that follow are the versions that were submitted during the peer review process. They are not the versions of record or the final published versions. They should not be cited or distributed as the published version of this manuscript.

BMJ Open is an open access journal and the full, final, typeset and author-corrected version of record of the manuscript is available on our site with no access controls, subscription charges or pay-per-view fees (http://bmjopen.bmj.com).

If you have any questions on BMJ Open's open peer review process please email editorial.bmjopen@bmj.com

Protocol for a randomised feasibility trial of a low intensity psychological intervention for depression in adults with autism: The Autism Depression Trial (ADEPT)

Journal:	*BMJ Open*
Manuscript ID	bmjopen-2017-019545
Article Type:	Protocol
Date Submitted by the Author:	11-Sep-2017

Complete List of Authors:	Russell, Ailsa; University of Bath, Psychology<br>Cooper, Kate; University of Bath, Psychology<br>Barton, stephen; Northumberland Tyne and Wear NHS Foundation Trust, Newcastle Cognitive and Behaviour Therapies Centre<br>Ensum, Ian; Avon and Wiltshire Mental Health Partnership NHS Trust, Bristol Adult Autism Services<br>Gaunt, Daisy; University of Bristol Faculty of Medicine and Dentistry, Bristol Randomised Trials Collaboration & School of Social and Community Medicine<br>Horwood, Jeremy; University of Bristol, UK, School of Social and Community Medicine<br>Ingham, Barry; Northumberland Tyne and Wear NHS Foundation Trust, Psychology<br>Kessler, David; University of Bristol School of Social and Community Medicine<br>Metcalfe, Chris; University of Bristol, Social Medicine<br>Rai, Dheeraj; University of Bristol, Centre for Academic Mental Health, School of Social & Community Medicine<br>Parr, Jeremy; Newcastle University, UK<br>Wiles, Nicola; Centre for Academic Mental Health, School of Social and Community Medicine, University of Bristol, Bristol, UK

**Primary Subject Heading**:	Mental health
Secondary Subject Heading:	Mental health, Evidence based practice
Keywords:	Autism, Depression & mood disorders < PSYCHIATRY, cognitive behaviour therapy, randomised controlled trial, low intensity intervention

SCHOLARONE™ Manuscripts

Protocol for a randomised feasibility trial of a low intensity psychological intervention for depression in adults with autism: The Autism Depression Trial (ADEPT)

Russell, A.J., Cooper, K., Barton, S.B., Ensum, I., Gaunt, D., Horwood, J., Ingham, B., Kessler, D., Metcalfe, C., Parr, J., Rai, D. and Wiles, N.

Ailsa Russell, Dept. of Psychology, University of Bath, Claverton Down, BA2 7AY
a.j.russell@bath.ac.uk
Kate Cooper, Dept. of Psychology, University of Bath, Claverton Down, BA2 7AY
K.Cooper@bath.ac.uk
Stephen Barton, Newcastle Cognitive and Behavioural Therapies Centre, Northumberland, Tyne & Wear NHS Trust, Stephen.barton@newcastle.ac.uk
Ian Ensum, Bristol Adult Autism Service, Avon &Wiltshire Mental Health Partnership NHS Trust
ian.ensum@nhs.net
Daisy Gaunt, Bristol Randomised Trials Collaboration, University of Bristol, Population Health Sciences, Bristol Medical School, Canynge Hall, 39 Whatley Road, Bristol BS8 2PS
daisy.gaunt@bristol.ac.uk
Jeremy Horwood, Bristol Randomised Trials Collaboration, University of Bristol, Canynge Hall, 39 Whatley Road, Bristol BS8 2PS j.horwood@bristol.ac.uk
Barry Ingham, Northumberland, Tyne & Wear NHS Trust, barry.ingham@ntw.nhs.uk
David Kessler, School of Social and Community Medicine, University of Bristol
david.kessler@bristol.ac.uk
Chris Metcalfe, Bristol Randomised Trials Collaboration, Canynge Hall, 39 Whatley Road, Bristol BS8 2PS chris.metcalfe@bristol.ac.uk
Dheeraj Rai, University of Bristol, Dheeraj.Rai@bristol.ac.uk
Jeremy Parr, Institute of Neuroscience, University of Newcastle upon Tyne, Jeremy.parr@ncl.ac.uk
Nicola Wiles, Centre for Academic Mental Health, University of Bristol Nicola.wiles@bristol.ac.uk

This project was funded by the Health Technology Assessment programme (HTA 14/43/02). This study was also supported by the NIHR Biomedical Research Centre at the University Hospitals Bristol NHS Foundation Trust and the University of Bristol.

Department of Health disclaimer

This report presents independent research commissioned by the National Institute for Health Research (NIHR). The views and opinions expressed by authors in this publication are those of the authors and do not necessarily reflect those of the NHS, the NIHR, MRC, CCF, NETSCC, the HTA programme or the Department of Health.

Other Acknowledgement: This study was designed and delivered in collaboration with the Bristol Randomised Trials Collaboration (BRTC), a UKCRC Registered Clinical Trials Unit in receipt of National Institute for Health Research CTU support funding. We would also like to acknowledge the support of the West of England and Northeast and North Cumbria Clinical Research Networks.

Authors Contributions: AR, DK, NW, SB, DR, JH, JP, BI and IE developed the study protocol, AR, SB and KC developed the intervention, NW provided methodological and statistical expertise in trial design, JH designed the qualitative evaluation, DG and CM developed the statistical analysis plan.

For peer review only - http://bmjopen.bmj.com/site/about/guidelines.xhtml

ABSTRACT

Introduction

Autism Spectrum Disorder (ASD) is a neurodevelopmental condition characterised by social communication impairments and repetitive behaviours. High rates of co-occurring depression have been reported in ASD. Cognitive behavioural interventions adapted for ASD have been shown to be effective in treating anxiety problems in this group. There have been several clinical evaluation studies of group CBT for co-occurring depression, but no randomised trials investigating low intensity psychological interventions as recommended in clinical guidelines for mild-moderate depression.

Methods and Analysis

A feasibility study comprising a randomised controlled trial (RCT) and nested qualitative evaluation is underway as preparation for a future definitive RCT. Participants (n=70) will be randomised to Guided Self-Help (GSH): a low intensity psychological intervention based on Behavioural Activation adapted for ASD or Treatment as Usual (TAU). Outcomes including depression symptoms, anxiety, social function, and service use will be measured at 10, 16 and 24 weeks post randomisation. Outcome measurement will be blind to group allocation for those measures that are not self-administered. The analysis will aim to establish the rates of recruitment and retention for a larger scale RCT as well as the most appropriate measure of depression to serve as primary outcome. The qualitative study will purposively sample up to 24 participants from each treatment group to consider the acceptability and feasibility of the intervention and the trial design.

Ethics and Dissemination

Approval to carry out the research has been received from the Health Research Authority with ethical approval from WALES REC 3 (IRAS project ID: 191558). There is a need for evidence based psychological interventions in people with autism and co-occurring depression. This feasibility study will consider if it is possible to conduct an effectiveness study of a newly developed, low intensity intervention for depression based on the principles of CBT for autistic adults.

Trial Registration: Current Controlled Trials ISRCTN54650760 assigned 28/09/2016

Keywords: Autism, Depression, Cognitive Behaviour Therapy, Low intensity intervention, Randomised Controlled Trial

Strengths and Limitations:

- This trial is funded following a commissioned call and thus aims to fill a clearly perceived clinical research and service gap
- Recruitment procedures ensure diagnostic validity of Autism Spectrum Disorder (ASD) and depression
- This is a feasibility trial and conclusions about effectiveness or efficacy of the intervention are not possible
- Including clinician as well as self-report of depression symptoms aims to overcome the documented difficulties with self-report of emotional states in ASD
- Patient and Public Involvement in the development of the intervention

For peer review only

For peer review only - http://bmjopen.bmj.com/site/about/guidelines.xhtml

BACKGROUND

Autism Spectrum Disorder (ASD) is a neurodevelopmental condition characterised by qualitative impairments in social communication and a pattern of restricted, repetitive behaviour, interests or activities[1]. High rates of co-occurring mental health problems have been reported in studies of children[2] and adults[3] with ASD. These include depression, a common mental disorder characterised by persistent low mood, feelings of guilt and low self-worth, an absence of positive affect and disturbances in sleep, appetite, concentration and energy levels. Depression is a debilitating mental health problem and has been estimated as the leading cause of loss of disability adjusted life years (DALYS) due to mental health and substance use disorders[4]. The estimated life-time point prevalence for a depressive episode for adults aged 16-74 in the U.K general population was 2.3% [5]. To date, there have been no robust epidemiological studies of depression in ASD but studies of clinical and research samples have reported high rates. For example a clinical study of 122 autistic adults with ASD found that 53% met criteria for depression at some point in their lives[6]. A recent systematic review reported rates of depression ranging from 4% to 35% across 7 studies of autistic adults without intellectual disability[7]. Although sampling and measurement methods may contribute to the lack of consistent findings across studies, there is some evidence to suggest that the prevalence of depression is notably higher in autistic adults than the general population.

The National Institute for Health and Care Excellence (NICE) Clinical Guidelines 90[8] recommend a stepped-care model for the treatment of depression. Mild-moderate depression should be treated according to Step 2 of the care pathway i.e. low intensity psychosocial interventions, psychological interventions, medication and referral for further assessment and treatment. Low-intensity interventions aim to increase access to evidence based psychological therapies. They are characterised by the provision of evidence based information, delivered by a range of 'technologies' such as written materials or computerised protocols, and facilitated by a mental health worker without formal professional training or self-facilitated. Monitoring and review are also important. Low intensity psychological interventions for depression as recommended by the clinical guidelines[8] are individual guided self-help based on the principles of cognitive behavioural therapy (CBT) to include behavioural activation and problem-solving techniques, computerised CBT (cCBT) or a structured group physical activity programme.

Cognitive Behavioural Therapy (CBT) refers to a group of psychological interventions which combine behavioural, cognitive and affect-focused techniques to bring about a reduction in psychological distress and an improvement in associated functional impairment. Adaptations to the delivery and content of CBT are outlined in the clinical guidance for adults with Autism[9]. Recent systematic reviews indicate that CBT can be effective in reducing anxiety problems in young people[10,11] and adults with ASD[12] if adapted. Meta-analysis of the studies of adapted CBT interventions for emotional disorders in children and adults report small-medium effect sizes (g= 0.24 on self-report outcomes, g= 0.66 on informant measures and g= 0.73 on clinician rated outcomes)[13].

Despite the high rates of co-occurring depression and the success in evaluating the effectiveness of adapting CBT for anxiety problems, there is a paucity of systematic evidence for its usefulness in treating depression in ASD. There have been 2 randomised evaluations of group CBT for young people with ASD using wait-list and cross-over trial designs[14,15] with mixed findings were reported in respect of changes on the primary outcome measures and relatively small sample sizes. An adult study[16] reported a significant effect of time but not treatment group on anxiety and

For peer review only - http://bmjopen.bmj.com/site/about/guidelines.xhtml

depression measures when comparing a CBT intervention with Mindfulness Based Stress Reduction (MBSR) both adapted for ASD. There are then suggestions that CBT adapted for ASD may be effective for adults with co-occurring depression, but to our knowledge, there have been no published clinical effectiveness studies of individual cognitive behavioural interventions, including Behavioural Activation or of a low intensity intervention.

Clinical trials aside, naturalistic treatment evaluations are less well described and reported. Thus, it is not known whether adults with ASD and co-occurring mild-moderate depression routinely access CBT interventions offered in primary care or experience less favourable treatment outcomes. It is known that the materials for low intensity CBT for depression have not been specifically developed with this group in mind.

Aims:

As part of a commissioned call by the NIHR HTA, this was a feasibility study for a large-scale Randomised Controlled Trial (RCT) that would determine the clinical and cost-effectiveness of a low intensity intervention for co-occurring depression in adults with autism. The aims of the feasibility study were to:

- Develop a low-intensity intervention for depression adapted for ASD based on NICE recommendations and accompanying training materials to guide therapists in supporting the intervention
- Investigate the feasibility and patient and therapist acceptability of the low intensity intervention.
- Estimate the rates of recruitment and retention for a large scale randomised controlled trial (RCT)
- Identify the most appropriate outcome measure for a large scale RCT

METHOD

Trial Design

The trial will comprise a single-blind, randomised controlled trial (RCT), with a nested qualitative evaluation. Participants will be randomly assigned to 1 of 2 treatment arms, a low-intensity intervention, Guided Self-Help for depression adapted for adults with Autism (GSH), or Treatment as Usual (TAU).

Recruitment

Participants (n=70) will be recruited from Adult Autism clinics (n=2) and research opportunities for adults with ASD (n=2).

Inclusion Criteria

- Aged ≥ 18 years
- Clinical diagnosis of Autism Spectrum Disorder (ASD)
- Current depression as measured by PHQ-9 score ≥ 10

For peer review only - http://bmjopen.bmj.com/site/about/guidelines.xhtml

Exclusion Criteria

- Participants who are non-English speaking
- Participants with literacy levels such that the written materials are inaccessible
- Risk of suicide such that a low intensity intervention is not in line with clinical need
- History of Psychosis
- Current alcohol/substance dependence
- Untreated epilepsy
- Attended > 6 sessions of individual CBT during the previous 6 months

Procedure

Clinicians in the NHS Adult Autism Clinics will introduce the study to potentially eligible participants i.e. where routine clinic assessment indicates current depression and the clinical risk assessment does not highlight increased risk of suicide or other risks which might mean a low intensity psychological intervention is not suitable.

Co-ordinators of the 2 research pathways will introduce the study to potentially eligible participants by mailshot, inviting people to find out more about the study according to the research opportunity protocols and guidelines.

Potentially eligible participants who express an interest in the study will be invited to contact the research team to find out more. Eligibility screening according to the inclusion and exclusion criteria will then be conducted by telephone. Participants meeting the inclusion/exclusion criteria will be invited to a meeting where eligibility will be confirmed using the Clinical Interview Schedule-Revised (CIS-R)[17], a standardised interview conducted by the researcher.

Fully informed, written consent to participate in the study will be sought. The researcher will ask participants to summarise in their own words what taking part in the study will involve, including enquiring about their understanding of the voluntary nature of the study.

Baseline assessment will be completed face to face for all consenting participants and include a number of self-report measures of depression, anxiety, obsessive compulsive symptoms, quality of life, social function, rumination, repetitive behaviours and a current use of services questionnaire (see Measures section). A clinician administered depression measure will also be completed, the Hamilton Rating Scale for Depression (HRSD).

A preferred communication and research contact strategy will be agreed with each individual at the outset of their participation in the study. As well as working with individual preferences, the aim is to minimise any retention issues occurring as a result of core characteristics of ASD. Individual preferences may involve carers or other family members, a sole reliance on electronic communication, repeated reminders etc.

Randomisation

Eligible and consenting participants will be randomly allocated to GSH (n=35) or TAU (n=35) via a remote computerised randomisation service. Randomisation will be stratified by NHS regional centre (n=2), and minimised by depression severity (mild-moderate, i.e. PHQ-9 scores between 10 and 15, or moderate-severe, i.e. PHQ-9 scores between 16 and 27) and anti-depressant medication

For peer review only - http://bmjopen.bmj.com/site/about/guidelines.xhtml

(currently taking/not taking). The trial manager will share details of the outcome of randomisation with the individual participants according to their communication preferences within 48 hours, and with the therapist. Follow-up measurement will be carried out by researchers who will remain blind to treatment allocation.

Figure 1 about here

Randomised treatments

Guided Self-Help

The intervention is based on the principles of Behavioural Activation for depression, adapted for ASD and delivered as a low intensity intervention.

The intervention will be delivered over 10 weeks. The first session (0) is a Planning session to orient the participant to the nature of guided self-help and the therapist to the nature of the participant's ASD. Individualised goals for treatment are formulated during this session. Sessions 1- 4 contain information and tasks to facilitate learning about the importance of links between situations, behaviour and feelings. Activity scheduling to increase opportunities for positive mood, in line with treatment goals, form the basis for sessions 5-8. Session materials are written with visual cues to enhance the accessibility of the text. A consistent session format is maintained throughout the treatment. Feedback and advice about the materials was obtained from 2 adults with ASD, both of whom had prior experience of CBT.

Therapist 'guides' will facilitate the sessions, encouraging participants to review and reflect on the session materials, and plan homework tasks. Therapist guides will be unqualified psychologists with a foundation knowledge in CBT consistent with the knowledge and skills of low-intensity therapists. They will include assistant psychologists and clinical psychologists in training. They will not ordinarily have the knowledge and training to deliver individual, formulation-driven CBT. Therapists will receive 15 hours of training in the intervention and 1 hour minimum of clinical supervision on a weekly basis. Supervision will be delivered by qualified clinical psychologists who have developed the intervention. It can be face to face or remote (via telephone/Skype) and can be delivered on an individual or group basis. A therapist manual has been developed for the intervention.

The intervention will be delivered face to face, with sessions lasting up to 45 minutes. The exception is the initial planning session which can last up to 90 minutes to facilitate engagement. The intervention can be delivered by telephone or Skype if it is otherwise inaccessible to a participant. However it is designed to be delivered face to face, with the latter sessions (5-8) amenable to delivery by telephone if preferred. Participants who attend ≥ 5 sessions will be considered to have received a minimum 'dose' of treatment.

Sessions will not be audio or video recorded. Therapists will complete a session record at the end of each session listing the treatment components covered in the session, engagement with the session, ease of completion of the session tasks and extent to which any homework tasks were completed. This will allow post-hoc evaluation of any logistical and pragmatic issues arising with the session materials. Progress with individual client treatment goals will be rated by the client on a scale of 0-10 in Sessions 0, 4 and 8.

For peer review only - http://bmjopen.bmj.com/site/about/guidelines.xhtml

Session by session measures will be completed, comprising the PHQ-9, GAD-7, brief Positive and Negative Affect Scale and engagement with daily activities. This will simulate routine practice in low intensity interventions where ongoing monitoring is an important element.

Treatment as Usual

There will be no restrictions on the care that can be provided as Treatment as Usual (TAU). Participants randomised to TAU will be provided with information about how to self-refer to local IAPT services. TAU may comprise no treatment, self or GP referral to IAPT services, Adult Autism clinic recommending GP referral to IAPT services, Adult Autism Clinic or GP referral to secondary mental health services, and/or anti-depressant medication. TAU will be carefully recorded in terms of the timing and nature of any intervention received.

Registration/Ethics

Approval to carry out the research has been received from the Health Research Authority with ethical approval from WALES REC 3 (IRAS project ID: 191558). R&D approval was received from Avon and Wiltshire Mental Health Partnership NHS Trust (AWP) and Northumberland, Tyne & Wear NHS Trust (NTW). The trial was registered on 28.09.2016 with the ISRCTN (Trial ID: ISRCTN54650760) http://www.isrctn.com/ISRCTN54650760

Eligibility Measure

Clinical Interview Schedule – Revised (CIS-R)[17,] The CIS-R is a structured diagnostic interview which generates ICD-10 psychiatric diagnoses. The CIS-R is a widely used, well validated diagnostic instrument. The CIS-R was administered via a computerised questionnaire in this study to assess the inclusion and exclusion criteria alongside the PHQ-9 score.

Outcome Measurement

Follow-up assessment will be conducted at 10, 16 and 24 weeks post-randomisation (See table 1 for a full schedule of assessment measures by time point). One aim of the study is to identify the most appropriate outcome measure for a large scale RCT, thus it is not possible to specify the primary outcome measure of depression a priori. The feasibility of delivering the intervention over 10 weeks will be evaluated. Thus it is not clear if 10 or 16 weeks post-randomisation will represent the most effective point of outcome measurement i.e. if the majority of participants are unable to complete the intervention within 10 weeks.

Depression Measures

The Patient Health Questionnaire (PHQ-9)[18] The PHQ-9 is a nine-item self-report measure of depression, which is commonly used in primary care settings. The PHQ-9 has been found to be reliable, valid, and sensitive to change in the general population (α=0.84-0.93)[19]. To the authors' knowledge the psychometric properties of the scale have not been investigated with the autistic population.

Beck Depression Inventory-II (BDI-II)[20] The BDI-II is a widely used, 21 -item self-report measure of depression. The BDI-II has been found to be reliable and valid, with a Cronbach's alpha of .92 for outpatients[20]. A validation study of 50 young people with ASD[21] reported good internal consistency on the BDI-II (Cronbach's alpha=0.90).

For peer review only - http://bmjopen.bmj.com/site/about/guidelines.xhtml

GRID-Hamilton Rating Scale for Depression (GRID-HAMD-17)[22] The GRID-HAMD is a version of the Hamilton Depression Rating Scale[23]. It is a 17-item clinician administered interview which has been found to be reliable and valid in the general population[22] but to our knowledge has not been investigated in the autistic population. GRID-HAMD interviews will be audio recorded with participant consent. Recordings will be independently rated by a second rater for all items (excluding items 8 and 9 which require face to face assessment i.e. observation of psychomotor retardation and agitation) to establish reliability of each assessor. This will be done for the first 6 interviews conducted by each assessor. To establish reliability across the study, a random sample (20%) of GRID-HAMD recordings will be independently rated.

Participant Global Rating of Change – Participants are asked to rate whether their depression is better, worse or much the same on a 5 point scale at 16 and 24 week follow-up.

Other Measures

General Anxiety Disorder Questionnaire (GAD-7)[24] The GAD-7 is a seven-item self-report measure of anxiety. The scale has been found to be reliable and valid in the typically developing population (Spitzer et al., 2006), with a Cronbach's alpha of 0.92, however its psychometric properties for autistic individuals is not known.

Positive and Negative Affect Schedule (PANAS)[25] The PANAS is a 20-item self-report scale of positive and negative affect. The scale has been found to be reliable and valid, with a Cronbach's alpha of .89 for the positive affect scale, and .85 for the negative affect scale[25] but has not been used with autistic individuals.

Obsessive Compulsive Inventory-Revised (OCI-R)[26] The OCI-R is an 18-item self-report measure of the symptoms of obsessive-compulsive disorder, with items such as "I feel I have to repeat certain numbers", which are scored on a five-point likert scale (0-4), indicating increasing frequency. The scale has been found to be reliable and valid (α=.81-.90)[26]. The OCI-R has been found to have good psychometric properties in a sample of 225 autistic people[27].

Work and Social Adjustment Scale (WSAS)[28] The WSAS is a five-item self-report measure of impaired functioning which has been found to be reliable and valid (α=0.7-0.94) in typically developing individuals, and although used with autism populations, its psychometric properties have not been assessed.

EQ-5D-5L [29] The EG-5D-5L is a five-item self-report measure of health, with items measured on a five-point likert scale (1-5) indicating severity. It has been found to be reliable and valid in the typically developing population[30].

12-Item Short Form Health Survey (SF-12)[31] The SF-12 is a 12-item self-report measure of physical and mental health . It has been found to be a reliable and valid measure among people with severe mental health problems[32], but psychometric properties for autistic populations are not available.

The Repetitive Behaviours Questionnaire (RBQ-2A)[33] The RBQ-2A is a 20 item self-report measure of repetitive behaviours. It has been found to have good internal consistency, with Cronbach alphas

For peer review only - http://bmjopen.bmj.com/site/about/guidelines.xhtml

For peer review only

between 0.73-0.83, and autistic people score significantly higher on the measure than typically developing individuals, suggesting that it is a valid measure of autism-specific repetitive behaviours.

The Rumination-Reflection Questionnaire (RRQ)[34] The RRQ is a 12-item self-report questionnaire, comprising 2 sub-scales; rumination and reflection. The measure has good psychometric properties, and the rumination scale has a cronbach's alpha of .90, and the reflection scale .91.

Economic evaluation - We will pilot the feasibility of data collection on statutory health and voluntary service use using a questionnaire collecting information on: use of other primary and community care services (NHS Direct, attendances at walk-in centres, use of community health care services); secondary care related to mental health (number of out-patient visits, type of clinic, and reason for visit; inpatient stays, length of stay and reason); use of social services and disability payments received; personal costs related to mental health (expenditure on over-the-counter medication, expenditure on prescriptions, travel costs associated with health care visits, loss of earnings, out of pocket expenditure on other services e.g. private counselling or complementary or alternative therapies, child care and domestic help); time off work and unpaid activities. We will also access GP records to provide information on: number of primary care consultations, by type e.g. face-to-face, telephone etc., and who seen; and prescribed medication.

Table 1.Timing of outcome measurement

Measure	Baseline	GSH Group Each Session	10 weeks(End of intervention)	16 weeks	24 weeks
Demographics	√				
PHQ-9	√	√	√	√	√
CIS-R	√				
BDI	√		√	√	√
SIGH-D	√		√	√	√
GAD-7	√	√	√	√	√
OCI-R	√		√	√	√
PANAS	√	√	√	√	√
SF-12	√		√	√	√
EQ-5D-5L	√		√	√	√
Participant Global Rating of Change				√	√
RBQ-2	√				
RRQ	√		√		
Economic evaluation			√	√	√

For peer review only - http://bmjopen.bmj.com/site/about/guidelines.xhtml

Statistical analysis

We will be following the recently published extension to CONSORT[35] when reporting the results of this feasibility trial. As this is a feasibility study there is no formal sample size calculation. Seventy participants was considered a large enough sample to consider the practicalities of recruitment and delivering the intervention.

For this feasibility study, we will calculate:

1. the proportion of adults with ASD consenting to the study;
2. the proportion completing the baseline assessment and entering the randomised phase;
3. for those in the intervention group, the number of guided self-help sessions attended and the proportion completing 5 or more sessions;
4. the proportion completing follow-up assessments at 10, 16 and 24 weeks post-randomisation.

We will explore the sensitivity to change of the various self-reported measures of depression in comparison with the GRID-HAMD (clinician rated assessment of depression) in order to identify the most appropriate outcome measures for the main trial. We will also compare the continuous scores on the depression outcome measures between groups. The standard deviation of the chosen primary outcome will inform the sample size calculation for the large-scale RCT.

We will evaluate the service use and economic evaluation questionnaire by examining the rates of completion across individual questions. We will compare the information provided by participants about primary and secondary care health service use with information gathered from GP and secondary care records.

Qualitative study

In-depth interviews will be conducted with participants (from both arms of the trial) 10 weeks after randomisation (after 10 week outcome measurement is complete). These interviews will consider and compare views and experiences of the trial and the acceptability of the guided self-help intervention. All therapists delivering the intervention will also be interviewed towards the end of the trial to illuminate the perceived effectiveness and acceptability of treatments and explore any barriers to its uptake outside of the trial.

Purposive sampling will select interviewees in order to attempt to capture maximum variation in views and experiences in order that they adequately reflect those of a range of participants. All participants in the trial will be asked if they are willing to be contacted about taking part in a qualitative interview at the time of trial consent. From participants who indicate that they are willing to be contacted, a purposive sample will be drawn in relation to (i) the trial site, (ii) arm of the trial and (iii) socio-demographic variables such as age, gender, ethnicity and socio-economic status (with participants being selected from areas of high and low social-economic deprivation, based on Index of Multiple Deprivation (IMD2007) score[36]. Interviews will be analysed in batches, and sampling will continue until no new themes are emerging from the interviews. This is likely to include up to 24 participants as well as 2-3 therapist interviews.

All interviews will be conducted by telephone or face-to-face in a location of the participants' choice. At interview, a flexible topic guide will be used to ensure primary issues are covered during all

For peer review only - http://bmjopen.bmj.com/site/about/guidelines.xhtml

interviews, but without dictating data collection, allowing participants to introduce unanticipated issues. Interviews are expected to last between 45-60 minutes. With informed consent, interviews will be recorded using a digital voice recorder, transcribed and anonymized.

Interview transcripts will be checked for accuracy and then imported into NVivo10 qualitative data analysis software[37], to aid management and indexing of data. Thematic analysis[38] utilising a data-driven inductive approach[39] will be used to scrutinise the data in order to identify and analyse patterns and themes of particular salience for participants and across the dataset using constant comparison techniques[40]. Analysis will begin shortly after data collection starts, will be ongoing and iterative. Analysis will inform further data collection: for instance, analytic insights from data gathered in earlier interviews will help identify any changes that need to be made to the topic guide during later interviews. Transcripts from the participants and therapists' interviews will be analysed separately, with coding frames being developed for each. A subset of transcripts will be independently double-coded by other members of the research team and compared. Discrepancies will be discussed and resolved to achieve a coding consensus.

Monitoring and adverse events

Adverse events and risk standardised operating procedures have been developed and will be followed by all researchers and therapists working on the trial. Adverse events are defined as significant negative episodes, or significant deterioration in condition, which happen to participants during their time in the trial. These will be reported by research assistants and trial therapists to senior trial staff, who will ascertain whether these are thought to be linked to participation in the trial, and keep records of each event on an adverse events database. Suicide risk will be monitored using the suicidality items on the PHQ-9 and BDI-II which are administered at baseline, and 10- 16- and 24-week follow-up. If the participant states that they have had suicidal/self-harming thoughts every day in the last week (PHQ-9) or that they would like to kill themselves, or would kill themselves if they had chance (BDI-II), then a letter will be sent to their GP highlighting the individual's risk. If the individual is considered to be high risk, a qualified clinician may also follow-up by offering information to the participant regarding local crisis teams, and call the GP to ensure the information is shared in a timely manner.

Independent Oversight

A Trial Steering Committee and Data Monitoring and Ethics Committee (DMEC) will have independent oversight of the study, meeting at 6 monthly intervals with quarterly reports of adverse and serious adverse events.

SUMMARY

High rates of co-occurring depression, a debilitating mental health problem, have been reported in adults with ASD[6]. There has been little research to date about the usefulness of adapted cognitive behaviour therapy for depression in autism or about routine service outcomes for this group. This feasibility study aims to develop a low intensity intervention for depression for adults with autism based on cognitive behavioural principles and accompanying therapist guidelines and training. The study design will enable the research team to consider the feasibility of carrying out a large-scale randomised controlled trial to consider the effectiveness of the intervention, including specifying the

For peer review only - http://bmjopen.bmj.com/site/about/guidelines.xhtml

For peer review only

most appropriate primary outcome measure. The nested qualitative study will address issues of patient and therapist acceptability of the intervention.

Trial status

The RCT is currently open to recruitment and the last participant is scheduled to be randomised by the end of September 2017. The study will run until the end of April 2018.

Competing Interests

Dr Russell has nothing to disclose.

REFERENCES

1 American Psychiatric Association Diagnostic and statistical manual of mental disorders (5th ed.) Washington, DC 2009.

2 Mannion, A, and Leader, G Co-morbidity in Autism Spectrum Disorder: A Review *Res Autism Spectr Disord* 2013; 7:1595-1616

3 Russell, AJ, Murphy, CM, Wilson, E et al. Short Report: The mental health of individuals referred for assessment of autism spectrum disorder in adulthood: A clinic report *Autism* 2016; **20(**5): 623-627

4 Whiteford HA, Degenhardt L, Rehm J, et al. Global burden of disease attributable to mental and substance use disorders: findings from the Global Burden of Disease Study 2010. *Lancet* 2013; **382**(9904): 1575-86.

5 McManus, S, Meltzer, H, Brugha, T, Bebbington, P. and Jenkins, R. (Editors) Adult Psychiatric Morbidity in England: results of a Household Survey The NHS Information Centre 2009

6 Hofvander, B, Delorme, R, Chaste, P, *et al.* Psychiatric and psychosocial problems in adults with normal-intelligence autism spectrum disorders *BMC Psychiatry* 2009; 9: 35; 1-9

7 Wigham S, Barton S, Parr JR et al. A systematic review of the rates of depression in children and adults with high functioning autism spectrum disorder *Journal of Mental Health Research in Intellectual Disabilities* 2017; **10**(4): 267-287.

8 NICE Clinical Guidelines 90 (CG90) *Depression in adults: recognition and management* National Institute for Healthcare and Excellence 2009

9 NICE Clinical Guidelines 142 (CG142)) *Autism Spectrum Disorder in adults: recognition and management* National Institute for Healthcare and Excellence 2012

10 Lang, R., Regester, A, Lauderdale, S et al. Treatment of anxiety in autism spectrum disorders using cognitive behaviour therapy: A systematic review *Developmental Rehabilitation* 2010; **13**(1): 53-63

11 Sukhodolsky, DG, Bloch, MH, Panza, KE et al. Cognitive behavioural therapy for anxiety in children with High-Functioning Autism: A Meta-analysis *Pediatrics* 2013; **132**(5):1341-1350

12 Spain, D, Sin, J, Chalder, T, et al. Cognitive behaviour therapy for adults with autism spectrum disorders and psychiatric co-morbidity: A Review *Res Autism Spectr Disord* 2015; 9: 151-162

13 Weston, L, Hodgekins, J, & Langdon, P E. Effectiveness of cognitive behavioural therapy with people who have autistic spectrum disorders: A systematic review and meta-analysis. *Clin Psychol Rev* 2016; 49: 41–54

14 Santomauro, D, Sheffield, J and Sofronoff, K Depression in adolescents with ASD: A pilot RCT of a group intervention *J Autism Dev Disord* 2016; **46**(2):572-588

For peer review only - http://bmjopen.bmj.com/site/about/guidelines.xhtml

15 McGilliveray, JA and Evert, HT Group cognitive behavioral program shows potential in reducing symptoms of depression and stress among young people with Autism Spectrum Disorders *J Autism Dev Disord* 2014; 44: 2041-51

16 Sizoo, BB and Kuiper, E. Cognitive Behaviour Therapy and Mindfulness Based Stress Reduction may be equally effective in reducing anxiety and depression in adults with Autism Spectrum Disorder *Res Dev Disabil* 2017; 64: 47-55

17 Lewis, G, Pelosi, AJ, Araya, R et al. Measuring psychiatric disorder in the community: a standardized assessment for use by lay interviewers. *Psychol Med* 1992; ***22***(2): 465-486.

18 Kroenke, K, & Spitzer, RL. The PHQ-9: a new depression diagnostic and severity measure. *Psychiatr Ann* 2002; ***32***(9):509-515

19 Cameron, I M, Crawford, JR, Lawton, K et al. Psychometric comparison of PHQ-9 and HADS for measuring depression severity in primary care *Br J Gen Pract* 2008; **58**(546): 32-36

20 Beck, A T, Steer, R A, & Brown, G K (1996) Beck Depression Inventory-II. *San Antonio*, 1996

21 Gotham, K, Unruh, K and Lord, C. Depression and its measurement in verbal adolescents and adults with autism spectrum disorder *Autism* 2015; **19**(4): 491-504

22 Williams, J B, Kobak, K A, Bech, P et al. The GRID-HAMD: standardization of the Hamilton depression rating scale. *Int Clin Psychopharmacol* 2008; ***23***(3):120-129.

23 Hamilton, M. (1960) A Rating Scale for Depression *J Neurol Neurosurg Psychiatry* 1960; 23: 56

24 Spitzer, R L, Kroenke, K, Williams, J B et al. A brief measure for assessing generalized anxiety disorder: the GAD-7 *Arch Intern Med* 2006;***166***(10):1092-1097.

25 Crawford, J R & Henry, J D The Positive and Negative Affect Schedule (PANAS): Construct validity, measurement properties and normative data in a large non-clinical sample *Br J Clin Psychol* 2004; ***43***(3): 245-265.

26 Foa, E B, Huppert, J D, Leiberg, S et al The Obsessive-Compulsive Inventory: development and validation of a short version *Psychol Assess* 2002; **14**(4): 485

27 Cadman, T, Spain, D, Johnston,P, et al Obsessive-compulsive disorder in adults with high-functioning autism spectrum disorder: what does self-report with the OCI-R tell us? *Autism Res* 2015; **8**(5): 477-85

28 Mundt, J C, Marks, I M, Shear, M K et al. The Work and Social Adjustment Scale: a simple measure of impairment in functioning *Br J Psychiatry* 2002;**180**(5):461-464

29 Herdman, M, Gudex, C, Lloyd, A et al. Development and preliminary testing of the new five-level version of EQ-5D (EQ-5D-5L). *Qual Life Res* 2011;***20***(10):1727-1736

30 Janssen, M F, Birnie, E, Haagsma, J A et al. (2008). Comparing the standard EQ-5D three-level system with a five-level version. *Value in Health* 2008;**11**(2):275-284

31 Ware, JE, Kosinski, M, and Keller, SD (1996) A 12-item short-form Health Survey (SF-12): Construction of scales and preliminary test of reliability *Medical Care* 1996; **32**(3): 220-233

32 Salyers, M P, Bosworth, H B, Swanson, J W et al. Reliability and validity of the SF-12 health survey among people with severe mental illness. *Medical care* 2000;**38**(11):1141-1150

33 Barrett, S L, Uljarević, M, Baker, E K et al. The Adult Repetitive Behaviours Questionnaire-2 (RBQ-2A): a self-report measure of restricted and repetitive behaviours. *J Autism Dev Disord* 2015;***45*****(**11): 3680-3692

34 Trapnell, P D, & Campbell, J D Private self-consciousness and the five-factor model of personality: distinguishing rumination from reflection. *J Pers Soc Psychol* 1999;**76**(2): 284

35 Eldridge, SM, Chan, CL. Campbell, MJ et al. CONSORT 2010 Statement: extension to randomised pilot and feasibility trials BMJ 2016; MJ,355:i5239 http://dx.doi.org/10.1136/bmj. i5239

For peer review only - http://bmjopen.bmj.com/site/about/guidelines.xhtml

36 Noble, M, McLennan, D, Wilkinson, K et al. The English Indices of Deprivation 2007 Communities and Local Government Publications; 2008

37 NVivo qualitative data analysis Software; QSR International Pty Ltd. Version 10, 2012

38 Braun V. & Clarke V. Using thematic analysis in psychology. *Qual Res Psychol* 2006;3:77-101

39 Boyatzis, R. *Transforming qualitative information: Thematic analysis and code development*. Thousand Oaks, CA: Sage 1998

40 Charmay K. *Constructing grounded theory: a practical guide through qualitative analysis*. London: Sage 2006

For peer review only

For peer review only - http://bmjopen.bmj.com/site/about/guidelines.xhtml

Figure 1. Timeline of events

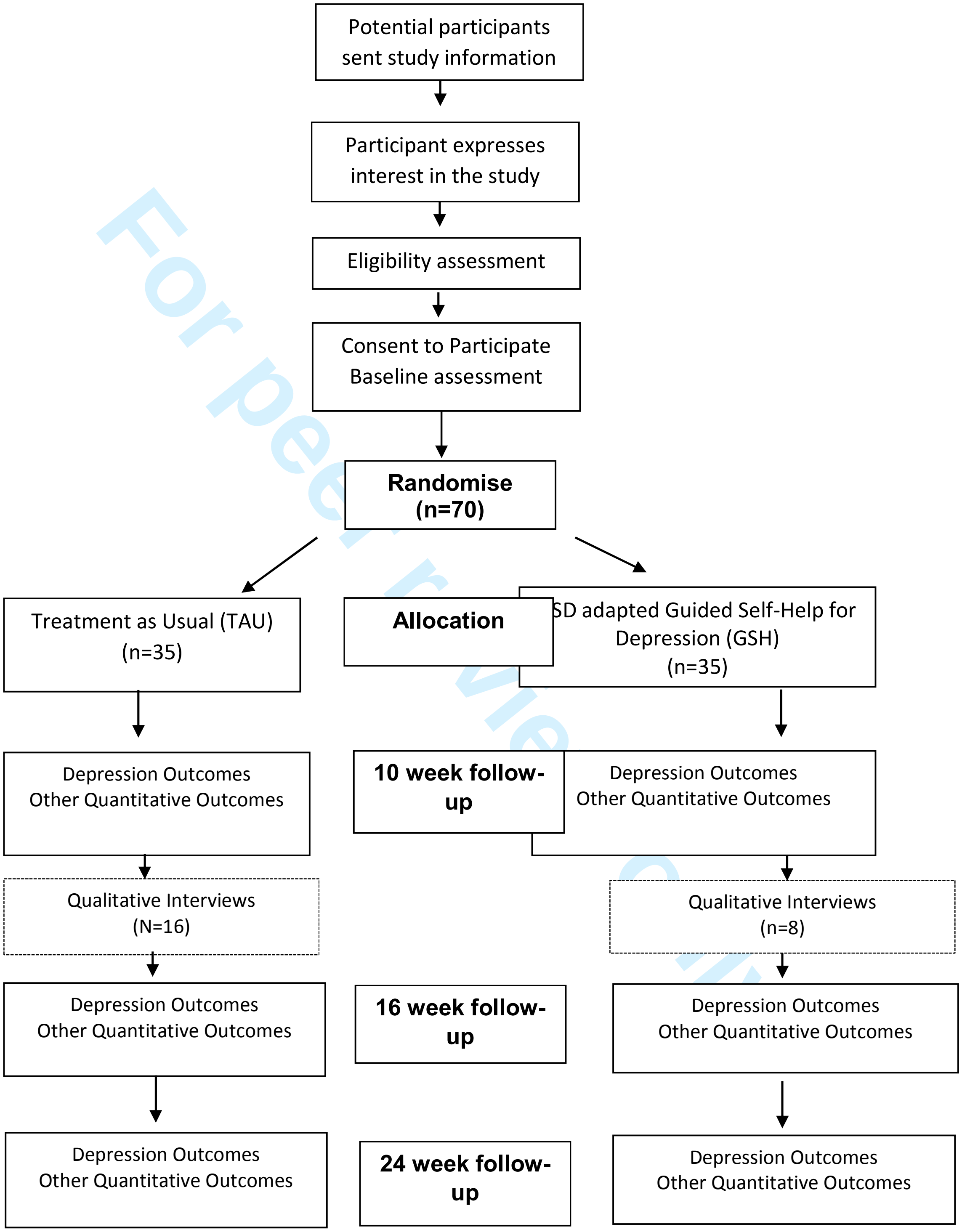

For peer review only - http://bmjopen.bmj.com/site/about/guidelines.xhtml

For peer review only

BMJ Open

Protocol for a feasibility study and randomised pilot trial of a low intensity psychological intervention for depression in adults with autism: The Autism Depression Trial (ADEPT)

Journal:	*BMJ Open*
Manuscript ID	bmjopen-2017-019545.R1
Article Type:	Protocol
Date Submitted by the Author:	29-Sep-2017
Complete List of Authors:	Russell, Ailsa; University of Bath, Psychology<br>Cooper, Kate; University of Bath, Psychology<br>Barton, stephen; Northumberland Tyne and Wear NHS Foundation Trust, Newcastle Cognitive and Behaviour Therapies Centre<br>Ensum, Ian; Avon and Wiltshire Mental Health Partnership NHS Trust, Bristol Adult Autism Services<br>Gaunt, Daisy; University of Bristol Faculty of Medicine and Dentistry, Bristol Randomised Trials Collaboration, Population Health Sciences,<br>Horwood, Jeremy; University of Bristol, UK, Bristol Randomised Trials Collaboration, Population Health Sciences, Bristol Medical School<br>Ingham, Barry; Northumberland Tyne and Wear NHS Foundation Trust, Psychology<br>Kessler, David; University of Bristol School of Social and Community Medicine<br>Metcalfe, Chris; University of Bristol, Bristol Randomised Trials Collaboration, Population Health Sciences, Bristol medical School<br>Rai, Dheeraj; University of Bristol, School of Social & Community Medicine<br>Parr, Jeremy; Newcastle University, UK, Institute of Neuroscience<br>Wiles, Nicola; Centre for Academic Mental Health, School of Social and Community Medicine, University of Bristol, Bristol, UK
**Primary Subject Heading**:	Mental health
Secondary Subject Heading:	Mental health, Evidence based practice
Keywords:	Autism, Depression & mood disorders < PSYCHIATRY, cognitive behaviour therapy, randomised controlled trial, low intensity intervention

SCHOLARONE™
Manuscripts

Protocol for a feasibility study and randomised pilot trial of a low intensity psychological intervention for depression in adults with autism: The Autism Depression Trial (ADEPT)

Russell, A.J., Cooper, K., Barton, S.B., Ensum, I., Gaunt, D., Horwood, J., Ingham, B., Kessler, D., Metcalfe, C., Parr, J., Rai, D. and Wiles, N.

Ailsa Russell, Dept. of Psychology[1], University of Bath, Claverton Down, BA2 7AY
a.j.russell@bath.ac.uk
Kate Cooper[1] K.Cooper@bath.ac.uk
Stephen Barton, Newcastle Cognitive and Behavioural Therapies Centre, Northumberland, Tyne & Wear NHS Trust, Stephen.barton@newcastle.ac.uk
Ian Ensum, Bristol Adult Autism Service, Avon &Wiltshire Mental Health Partnership NHS Trust
ian.ensum@nhs.net
Daisy Gaunt[2] Bristol Randomised Trials Collaboration, Population Health Sciences, Bristol Medical School, University of Bristol, Canynge Hall, 39 Whatley Road, Bristol BS8 2PS
daisy.gaunt@bristol.ac.uk
Jeremy Horwood[2] j.horwood@bristol.ac.uk
Barry Ingham, Northumberland, Tyne & Wear NHS Trust, barry.ingham@ntw.nhs.uk
David Kessler[3] School of Social and Community Medicine, Bristol Medical School, University of Bristol
david.kessler@bristol.ac.uk
Chris Metcalfe [2] chris.metcalfe@bristol.ac.uk
Dheeraj Rai [3] Dheeraj.Rai@bristol.ac.uk
Jeremy Parr, Institute of Neuroscience, University of Newcastle upon Tyne, Jeremy.parr@ncl.ac.uk
Nicola Wiles, Centre for Academic Mental Health, Population Health Sciences, Bristol Medical School, University of Bristol Nicola.wiles@bristol.ac.uk

This project was funded by the Health Technology Assessment programme (HTA 14/43/02). This study was also supported by the NIHR Biomedical Research Centre at the University Hospitals Bristol NHS Foundation Trust and the University of Bristol.

Department of Health disclaimer

This report presents independent research commissioned by the National Institute for Health Research (NIHR). The views and opinions expressed by authors in this publication are those of the authors and do not necessarily reflect those of the NHS, the NIHR, MRC, CCF, NETSCC, the HTA programme or the Department of Health.

Other Acknowledgement: This study was designed and delivered in collaboration with the Bristol Randomised Trials Collaboration (BRTC), a UKCRC Registered Clinical Trials Unit in receipt of National Institute for Health Research CTU support funding. We would also like to acknowledge the support of the West of England and Northeast and North Cumbria Clinical Research Networks.

Authors Contributions: AR, DK, NW, SB, DR, JH, JP, BI and IE developed the study protocol, AR, SB and KC developed the intervention, NW provided methodological and statistical expertise in trial design, JH designed the qualitative evaluation, DG and CM developed the statistical analysis plan.

For peer review only - http://bmjopen.bmj.com/site/about/guidelines.xhtml

ABSTRACT

Introduction

High rates of co-occurring depression are reported in Autism Spectrum Disorder (ASD), a neurodevelopmental condition characterised by social communication impairments and repetitive behaviours. Cognitive behavioural interventions adapted for ASD have been effective for anxiety problems. There have been evaluation studies of group CBT for co-occurring depression, but no randomised trials investigating low intensity psychological interventions as recommended in clinical guidelines for mild-moderate depression.

Methods and Analysis

A feasibility study comprising a randomised controlled trial (RCT) and nested qualitative evaluation is underway as preparation for a definitive RCT. Participants (n=70) will be randomised to Guided Self-Help (GSH): a low intensity psychological intervention based on Behavioural Activation adapted for ASD or Treatment as Usual (TAU). Outcomes including depression symptoms, anxiety, social function, and service use will be measured at 10, 16 and 24 weeks post randomisation and will be blind to group allocation for measures that are not self-administered. The analysis will aim to establish the rates of recruitment and retention for a larger scale RCT as well as the most appropriate measure of depression to serve as primary outcome. The qualitative study will purposively sample up to 24 participants from each treatment group to consider the acceptability and feasibility of the intervention and the trial design.

Ethics and Dissemination

Ethical approval has been received from WALES REC 3 (IRAS project ID: 191558) and the Health Research Authority with R&D approval from Avon and Wiltshire Mental Health Partnership (AWP) and Northumberland, Tyne & Wear (NTW) NHS Trusts To our knowledge, this is the first study of a low intensity intervention for depression in adults with Autism. The results will inform the design of a definitive RCT. Dissemination will include peer-reviewed journal publications reporting the quantitative and qualitative research findings of the study, and presentations at national and international conferences.

Trial Registration: Current Controlled Trials ISRCTN54650760 assigned 28/09/2016 http://www.isrctn.com/ISRCTN54650760

Keywords: Autism, Depression, Cognitive Behaviour Therapy, Low intensity intervention, Randomised Controlled Trial

Strengths and Limitations:

- This trial is funded following a commissioned call and thus aims to fill a clearly perceived clinical research and service gap
- Recruitment procedures ensure diagnostic validity of Autism Spectrum Disorder (ASD) and depression
- Conclusions about effectiveness or efficacy of the intervention are not possible
- Inclusion of clinician as well as self-report measures of depression symptoms aims to overcome the documented difficulties with self-report of emotional states in ASD
- Patient and Public Involvement in the development of the intervention

For peer review only - http://bmjopen.bmj.com/site/about/guidelines.xhtml

BACKGROUND

Autism Spectrum Disorder (ASD) is a neurodevelopmental condition characterised by qualitative impairments in social communication and a pattern of restricted, repetitive behaviour, interests or activities[1]. High rates of co-occurring mental health problems have been reported in studies of children[2] and adults[3] with ASD. These include depression, a common mental disorder characterised by persistent low mood, feelings of guilt and low self-worth, an absence of positive affect and disturbances in sleep, appetite, concentration and energy levels. Depression is a debilitating mental health problem and has been estimated as the leading cause of loss of disability adjusted life years (DALYS) due to mental health and substance use disorders[4]. The estimated life-time prevalence for a depressive episode for adults aged 16-74 in the U.K general population was 2.3% [5]. To date, there have been no robust epidemiological studies of depression in ASD but studies of clinical and research samples have reported high rates. For example a clinical study of 122 autistic adults with ASD found that 53% met criteria for depression at some point in their lives[6]. A recent systematic review reported rates of depression ranging from 4% to 35% across 7 studies of autistic adults without intellectual disability[7]. Although sampling and measurement methods may contribute to the lack of consistent findings across studies, there is some evidence to suggest that the prevalence of depression is notably higher in autistic adults than the general population.

The National Institute for Health and Care Excellence (NICE) Clinical Guidelines 90[8] recommend a stepped-care model for the treatment of depression. Mild-moderate depression should be treated according to Step 2 of the care pathway i.e. low intensity psychosocial interventions, psychological interventions, medication and referral for further assessment and treatment. Low-intensity interventions aim to increase access to evidence based psychological therapies. They are characterised by the provision of evidence based information, delivered by a range of 'technologies' such as written materials or computerised protocols, and facilitated by a mental health worker without formal professional training or self-facilitated. Monitoring and review are also important. Low intensity psychological interventions for depression as recommended by the clinical guidelines[8] are individual guided self-help based on the principles of cognitive behavioural therapy (CBT) to include behavioural activation and problem-solving techniques, computerised CBT (cCBT) or a structured group physical activity programme.

Cognitive Behavioural Therapy (CBT) refers to a group of psychological interventions which combine behavioural, cognitive and affect-focused techniques to bring about a reduction in psychological distress and an improvement in associated functional impairment. Adaptations to the delivery and content of CBT are outlined in the clinical guidance for adults with Autism[9]. Recent systematic reviews indicate that CBT can be effective in reducing anxiety problems in young people[10,11] and adults with ASD[12] if adapted. Meta-analysis of the studies of adapted CBT interventions for emotional disorders in children and adults report small-medium effect sizes (g= 0.24 on self-report outcomes, g= 0.66 on informant measures and g= 0.73 on clinician rated outcomes)[13].

Despite the high rates of co-occurring depression and the success in evaluating the effectiveness of adapting CBT for anxiety problems, there is a paucity of systematic evidence for its usefulness in treating depression in ASD. There have been 2 randomised evaluations of group CBT for young people with ASD using wait-list and cross-over trial designs[14,15] with mixed findings reported in respect of changes on the primary outcome measures and relatively small sample sizes. An adult study[16] reported a significant effect of time but not treatment group on anxiety and depression

For peer review only - http://bmjopen.bmj.com/site/about/guidelines.xhtml

measures when comparing a CBT intervention with Mindfulness Based Stress Reduction (MBSR) both adapted for ASD. There are then suggestions that CBT adapted for ASD may be effective for adults with co-occurring depression, but to our knowledge, there have been no published clinical effectiveness studies of individual cognitive behavioural interventions, including Behavioural Activation or of a low intensity intervention.

Clinical trials aside, naturalistic treatment evaluations are less well described and reported. Thus, it is not known whether adults with ASD and co-occurring mild-moderate depression routinely access CBT interventions offered in primary care or experience less favourable treatment outcomes. It is known that the materials for low intensity CBT for depression have not been specifically developed with this group in mind.

Aims:

As part of a commissioned call by the NIHR HTA, this was a feasibility study for a large-scale Randomised Controlled Trial (RCT) that would determine the clinical and cost-effectiveness of a low intensity intervention for co-occurring depression in adults with autism. The aims of the feasibility study were to:

- Develop a low-intensity intervention for depression adapted for ASD based on NICE recommendations and accompanying training materials to guide therapists in supporting the intervention
- Investigate the feasibility and patient and therapist acceptability of the low intensity intervention.
- Estimate the rates of recruitment and retention for a large scale randomised controlled trial (RCT)
- Identify the most appropriate outcome measure for a large scale RCT

METHOD

Trial Design

The trial will comprise a single-blind, randomised controlled trial (RCT), with a nested qualitative evaluation. Participants will be randomly assigned to 1 of 2 treatment arms, a low-intensity intervention, Guided Self-Help for depression adapted for adults with Autism (GSH), or Treatment as Usual (TAU).

Recruitment

Participants (n=70) will be recruited from Adult Autism clinics (n=2) and research organisations for adults with ASD (n=2).

Inclusion Criteria

- Aged ≥ 18 years
- Clinical diagnosis of Autism Spectrum Disorder (ASD)
- Current depression as measured by PHQ-9 score ≥ 10

For peer review only - http://bmjopen.bmj.com/site/about/guidelines.xhtml

For peer review only

Exclusion Criteria

- Participants who are non-English speaking
- Participants with literacy levels such that the written materials are inaccessible
- Risk of suicide such that a low intensity intervention is not in line with clinical need
- History of Psychosis
- Current alcohol/substance dependence
- Untreated epilepsy
- Attended > 6 sessions of individual CBT during the previous 6 months

Procedure

Clinicians in the NHS Adult Autism Clinics will introduce the study to potentially eligible participants.

Co-ordinators of the 2 research pathways will introduce the study to potentially eligible participants by mailshot, inviting people to find out more about the study according to the research opportunity protocols and guidelines.

Potentially eligible participants who express an interest in the study will be invited to contact the research team to find out more. Eligibility screening according to the inclusion and exclusion criteria will then be conducted by telephone. Participants meeting the inclusion/exclusion criteria will be invited to a meeting where eligibility will be confirmed using the Clinical Interview Schedule-Revised (CIS-R)[17], a standardised interview conducted by the researcher.

Fully informed, written consent to participate in the study will be sought. The researcher will ask participants to summarise in their own words what taking part in the study will involve, including enquiring about their understanding of the voluntary nature of the study.

Baseline assessment will be completed face to face for all consenting participants and include a number of self-report measures of depression, anxiety, obsessive compulsive symptoms, quality of life, social function, rumination, repetitive behaviours and a current use of services questionnaire (see Measures section). A clinician administered depression measure will also be completed, the Hamilton Rating Scale for Depression (HRSD).

A preferred communication and research contact strategy will be agreed with each individual at the outset of their participation in the study. As well as working with individual preferences, the aim is to minimise any retention issues occurring as a result of core characteristics of ASD. Individual preferences may involve carers or other family members, a sole reliance on electronic communication, repeated reminders etc.

Randomisation

Eligible and consenting participants will be randomly allocated to GSH (n=35) or TAU (n=35) via a remote computerised randomisation service. Randomisation will be stratified by NHS regional centre (n=2), and minimised by depression severity (mild-moderate, i.e. PHQ-9 scores between 10 and 15, or moderate-severe, i.e. PHQ-9 scores between 16 and 27) and anti-depressant medication (currently taking/not taking). The trial manager will share details of the outcome of randomisation with the individual participants according to their communication preferences within 48 hours, and

For peer review only - http://bmjopen.bmj.com/site/about/guidelines.xhtml

with the therapist. Follow-up measurement will be carried out by researchers who will remain blind to treatment allocation (See Figure 1 for timeline of events).

Figure 1 about here

Randomised treatments

Guided Self-Help

The intervention is based on the principles of Behavioural Activation for depression, adapted for ASD and delivered as a low intensity intervention.

The intervention will be delivered over 10 weeks. The first session (0) is a Planning session to orient the participant to the nature of guided self-help and the therapist to the nature of the participant's ASD. Individualised goals for treatment are formulated during this session. Sessions 1- 4 contain information and tasks to facilitate learning about the importance of links between situations, behaviour and feelings. Activity scheduling to increase opportunities for positive mood, in line with treatment goals, form the basis for sessions 5-8. Session materials are written with visual cues to enhance the accessibility of the text. A consistent session format is maintained throughout the treatment. Feedback and advice about the materials during development was obtained from 2 adults with ASD. They provided feedback about the clarity and acceptability of the written text, accompanying visual images, suggested homework tasks, the content of examples used to convey concepts, the visual layout and format of the written materials, and the amount of material provided for each individual session. A focus group of adults with ASD was then asked to provide feedback on the final version of the Planning session and the study information sheets.

Therapist 'guides' will facilitate the sessions, encouraging participants to review and reflect on the session materials, and plan homework tasks. Therapist guides will be unqualified psychologists with a foundation knowledge in CBT consistent with the knowledge and skills of low-intensity therapists. They will include assistant psychologists and clinical psychologists in training. They will not ordinarily have the knowledge and training to deliver individual, formulation-driven CBT. Therapists will receive 15 hours of training in the intervention and 1 hour minimum of clinical supervision on a weekly basis. Supervision will be delivered by qualified clinical psychologists who have developed the intervention. It can be face to face or remote (via telephone/Skype) and can be delivered on an individual or group basis. A therapist manual has been developed for the intervention.

The intervention will be delivered face to face, with sessions lasting up to 45 minutes. The exception is the initial planning session which can last up to 90 minutes to facilitate engagement. The intervention can be delivered by telephone or Skype if it is otherwise inaccessible to a participant. However it is designed to be delivered face to face, with the latter sessions (5-8) amenable to delivery by telephone if preferred. Participants who attend ≥ 5 sessions will be considered to have received a minimally effective dose of treatment.

Sessions will not be audio or video recorded. Therapists will complete a session record at the end of each session listing the treatment components covered in the session, engagement with the session, ease of completion of the session tasks and extent to which any homework tasks were completed. This will allow post-hoc evaluation of any logistical and pragmatic issues arising with the session

For peer review only - http://bmjopen.bmj.com/site/about/guidelines.xhtml

materials. Progress with individual client treatment goals will be rated by the client on a scale of 0-10 in Sessions 0, 4 and 8.

Session by session measures will be completed, comprising the PHQ-9, GAD-7, brief Positive and Negative Affect Scale and engagement with daily activities. This will simulate routine practice in low intensity interventions where ongoing monitoring is an important element.

Treatment as Usual

There will be no restrictions on the care that can be provided as Treatment as Usual (TAU). Participants randomised to TAU will be provided with information about how to self-refer to local IAPT services. TAU may comprise no treatment, self or GP referral to IAPT services, Adult Autism clinic recommending GP referral to IAPT services, Adult Autism Clinic or GP referral to secondary mental health services, and/or anti-depressant medication. TAU will be carefully recorded in terms of the timing and nature of any intervention received.

Eligibility Measure

Clinical Interview Schedule – Revised (CIS-R)[17,] The CIS-R is a structured diagnostic interview which generates ICD-10 psychiatric diagnoses. The CIS-R is a widely used, well validated diagnostic instrument. The CIS-R was administered via a computerised questionnaire in this study to assess the inclusion and exclusion criteria alongside the PHQ-9 score.

Outcome Measurement

Follow-up assessment will be conducted at 10, 16 and 24 weeks post-randomisation (See table 1 for a full schedule of assessment measures by time point) and will be carried out by researchers blind to treatment allocation. One aim of the study is to identify the most appropriate outcome measure for a large scale RCT, thus it is not possible to specify the primary outcome measure of depression a priori. The feasibility of delivering the intervention over 10 weeks will be evaluated. Thus it is not clear if 10 or 16 weeks post-randomisation will represent the most effective point of outcome measurement i.e. if the majority of participants are unable to complete the intervention within 10 weeks.

Depression Measures

The Patient Health Questionnaire (PHQ-9)[18] The PHQ-9 is a nine-item self-report measure of depression, which is commonly used in primary care settings. The PHQ-9 has been found to be reliable (Cronbach's α=0.84-0.93), valid, and sensitive to change in the general population [19]. To the authors' knowledge the psychometric properties of the scale have not been investigated with the autistic population.

Beck Depression Inventory-II (BDI-II)[20] The BDI-II is a widely used, 21 -item self-report measure of depression. The BDI-II has been found to be reliable (Cronbach's α=0 .92) for outpatients[20]. A validation study of 50 young people with ASD[21] reported good internal consistency on the BDI-II (Cronbach's alpha=0.90).

GRID-Hamilton Rating Scale for Depression (GRID-HAMD-17)[22] The GRID-HAMD is a version of the Hamilton Depression Rating Scale[23]. It is a 17-item clinician administered interview which has been found to be reliable and valid in the general population[22] but to our knowledge has not been

For peer review only - http://bmjopen.bmj.com/site/about/guidelines.xhtml

investigated in the autistic population. GRID-HAMD interviews will be audio recorded with participant consent. Recordings will be independently rated by a second rater for all items (excluding items 8 and 9 which require face to face assessment i.e. observation of psychomotor retardation and agitation) to establish reliability of each assessor. This will be done for the first 6 interviews conducted by each assessor. To establish reliability across the study, a random sample (20%) of GRID-HAMD recordings will be independently rated.

Participant Global Rating of Change – Participants are asked to rate whether their depression is better, worse or much the same on a 5 point scale at 16 and 24 week follow-up.

Other Measures

General Anxiety Disorder Questionnaire (GAD-7)[24] The GAD-7 is a seven-item self-report measure of anxiety. The scale has been found to be reliable and valid in the typically developing population [24], (Cronbach's α=0.92). However the psychometric properties for use with autistic individuals is not known.

Positive and Negative Affect Schedule (PANAS)[25] The PANAS is a 20-item self-report scale of positive and negative affect. The scale has been found to be reliable with a Cronbach's alpha of .89 for the positive affect scale, and .85 for the negative affect scale[25] but has not been used with autistic individuals.

Obsessive Compulsive Inventory-Revised (OCI-R)[26] The OCI-R is an 18-item self-report measure of the symptoms of obsessive-compulsive disorder, with items such as "I feel I have to repeat certain numbers", which are scored on a five-point likert scale (0-4), indicating increasing frequency. The scale has been found to be reliable and valid[26]. The OCI-R has been found to have good psychometric properties in a sample of 225 autistic people[27].

Work and Social Adjustment Scale (WSAS)[28] The WSAS is a five-item self-report measure of impaired functioning which has been found to be reliable (Cronbach's α=0.7-0.94) and valid in typically developing individuals. It has been used with autism populations, but its psychometric properties have not been assessed.

EQ-5D-5L [29] The EG-5D-5L is a five-item self-report measure of health, with items measured on a five-point likert scale (1-5) indicating severity. It has been found to be reliable and valid in the typically developing population[30].

12-Item Short Form Health Survey (SF-12)[31] The SF-12 is a 12-item self-report measure of physical and mental health . It has been found to be a reliable and valid measure among people with severe mental health problems[32], but psychometric properties for autistic populations are not available.

The Repetitive Behavours Questionnaire (RBQ-2A)[33] The RBQ-2A is a 20 item self-report measure of repetitive behaviours. It has been found to have good internal consistency, with Cronbach alphas between 0.73-0.83, and autistic people score significantly higher on the measure than typically developing individuals, suggesting that it is a valid measure of autism-specific repetitive behaviours.

For peer review only

For peer review only - http://bmjopen.bmj.com/site/about/guidelines.xhtml

The Rumination-Reflection Questionnaire (RRQ)[34] The RRQ is a 12-item self-report questionnaire, comprising 2 sub-scales; rumination and reflection. The measure has good psychometric properties, and the rumination scale has a cronbach's alpha of .90, and the reflection scale .91.

Economic evaluation - We will pilot the feasibility of data collection on statutory health and voluntary service use using a questionnaire collecting information on: use of other primary and community care services (NHS Direct, attendances at walk-in centres, use of community health care services); secondary care related to mental health (number of out-patient visits, type of clinic, and reason for visit; inpatient stays, length of stay and reason); use of social services and disability payments received; personal costs related to mental health (expenditure on over-the-counter medication, expenditure on prescriptions, travel costs associated with health care visits, loss of earnings, out of pocket expenditure on other services e.g. private counselling or complementary or alternative therapies, child care and domestic help); time off work and unpaid activities. We will also access GP records to provide information on: number of primary care consultations, by type e.g. face-to-face, telephone etc., and who seen; and prescribed medication.

Table 1.Timing of outcome measurement

Measure	Baseline	GSH Group Each Session	10 weeks(End of intervention)	16 weeks	24 weeks
Demographics	√				
PHQ-9	√	√	√	√	√
CIS-R	√				
BDI	√		√	√	√
SIGH-D	√		√	√	√
GAD-7	√	√	√	√	√
OCI-R	√		√	√	√
PANAS	√	√	√	√	√
SF-12	√		√	√	√
EQ-5D-5L	√		√	√	√
Participant Global Rating of Change				√	√
RBQ-2	√				
RRQ	√		√		
Economic evaluation			√	√	√

For peer review only - http://bmjopen.bmj.com/site/about/guidelines.xhtml

Statistical analysis

We will be following the recently published extension to CONSORT[35] when reporting the results of this feasibility trial. As this is a feasibility study there is no formal sample size calculation. Seventy participants was considered a large enough sample to consider the practicalities of recruitment and delivering the intervention.

For this feasibility study, we will calculate and present in a CONSORT flow-chart:

1. the proportion of adults with ASD consenting to the study;
2. the proportion completing the baseline assessment and entering the randomised phase;
3. for those in the intervention group, the number of guided self-help sessions attended and the proportion completing 5 or more sessions;
4. the proportion completing follow-up assessments at 10, 16 and 24 weeks post-randomisation.

We will explore the sensitivity to change of the various self-reported measures of depression in comparison with the GRID-HAMD (clinician rated assessment of depression) in order to identify the most appropriate outcome measures for the main trial. We will also compare the continuous scores on the depression outcome measures between groups. The standard deviation of the chosen primary outcome will inform the sample size calculation for the large-scale RCT.

We will evaluate the service use and economic evaluation questionnaire by examining the rates of completion across individual questions. We will compare the information provided by participants about primary and secondary care health service use with information gathered from GP and secondary care records.

Qualitative study

In-depth interviews will be conducted with participants (from both arms of the trial) 10 weeks after randomisation (after 10 week outcome measurement is complete). These interviews will consider and compare views and experiences of the trial and the acceptability of the guided self-help intervention. All therapists delivering the intervention will also be interviewed towards the end of the trial to illuminate the perceived effectiveness and acceptability of treatments and explore any barriers to its uptake outside of the trial.

Purposive sampling will select interviewees in order to attempt to capture maximum variation in views and experiences in order that they adequately reflect those of a range of participants. All participants in the trial will be asked if they are willing to be contacted about taking part in a qualitative interview at the time of trial consent. From participants who indicate that they are willing to be contacted, a purposive sample will be drawn in relation to (i) the trial site, (ii) arm of the trial and (iii) socio-demographic variables such as age, gender, ethnicity and socio-economic status (with participants being selected from areas of high and low social-economic deprivation, based on Index of Multiple Deprivation (IMD2007) score[36]. Interviews will be analysed in batches, and sampling will continue until no new themes are emerging from the interviews. This is likely to include up to 24 participants as well as 2-3 therapist interviews.

All interviews will be conducted by telephone or face-to-face in a location of the participants' choice. At interview, a flexible topic guide will be used to ensure primary issues are covered during all

For peer review only - http://bmjopen.bmj.com/site/about/guidelines.xhtml

interviews, but without dictating data collection, allowing participants to introduce unanticipated issues. Interviews are expected to last between 45-60 minutes. With informed consent, interviews will be recorded using a digital voice recorder, transcribed and anonymized.

Interview transcripts will be checked for accuracy and then imported into NVivo10 qualitative data analysis software[37], to aid management and indexing of data. Thematic analysis[38] utilising a data-driven inductive approach[39] will be used to scrutinise the data in order to identify and analyse patterns and themes of particular salience for participants and across the dataset using constant comparison techniques[40]. Analysis will begin shortly after data collection starts, will be ongoing and iterative. Analysis will inform further data collection: for instance, analytic insights from data gathered in earlier interviews will help identify any changes that need to be made to the topic guide during later interviews. Transcripts from the participants and therapists' interviews will be analysed separately, with coding frames being developed for each. A subset of transcripts will be independently double-coded by other members of the research team and compared. Discrepancies will be discussed and resolved to achieve a coding consensus.

Monitoring and adverse events

Adverse events and risk standardised operating procedures have been developed and will be followed by all researchers and therapists working on the trial. Adverse events are defined as significant negative episodes, or significant deterioration in condition, which happen to participants during their time in the trial. These will be reported by research assistants and trial therapists to senior trial staff, who will ascertain whether these are thought to be linked to participation in the trial, and keep records of each event on an adverse events database. All serious adverse events of an unexpected and unrelated nature will be reported to the main Research Ethics Committee, the study Sponsor and Trial Steering Committee (TSC). Suicide risk will be monitored using the suicidality items on the PHQ-9 and BDI-II which are administered at baseline, and 10- 16- and 24-week follow-up. If the participant states that they have had suicidal/self-harming thoughts every day in the last week (PHQ-9) or that they would like to kill themselves, or would kill themselves if they had chance (BDI-II), then a letter will be sent to their GP highlighting the individual's risk. If the individual is considered to be high risk, a qualified clinician may also follow-up by offering information to the participant regarding local crisis teams, and call the GP to ensure the information is shared in a timely manner.

Independent Oversight

A Trial Steering Committee and Data Monitoring and Ethics Committee (DMEC) will have independent oversight of the study, meeting at 6 monthly intervals with quarterly reports of adverse and serious adverse events.

Potential Limitations

The majority of participants in this study will receive a diagnosis of Autism Spectrum Disorder (ASD) during adulthood and thus may not be representative of all adults with ASD.

Treatment as Usual (TAU) may vary considerably by geographical region and service factors may act as a potential confound.

SUMMARY

For peer review only - http://bmjopen.bmj.com/site/about/guidelines.xhtml

High rates of co-occurring depression, a debilitating mental health problem, have been reported in adults with ASD[6]. There has been little research to date about the usefulness of adapted cognitive behaviour therapy for depression in autism or about routine service outcomes for this group. This feasibility study aims to develop a low intensity intervention for depression for adults with autism based on cognitive behavioural principles and accompanying therapist guidelines and training. The study design will enable the research team to consider the feasibility of carrying out a large-scale randomised controlled trial to consider the effectiveness of the intervention, including specifying the most appropriate primary outcome measure. The nested qualitative study will address issues of patient and therapist acceptability of the intervention.

Trial status

The pilot RCT is currently open to recruitment and the last participant is scheduled to be randomised by the end of September 2017. The trial will run until the end of April 2018.

The full trial protocol can be accessed at
https://www.journalslibrary.nihr.ac.uk/programmes/hta/144302#/

Competing Interests

Drs Cooper, Ensum, Gaunt, Horwood, Kessler, Parr, Rai and Russell have nothing to disclose; Dr Barton reports grants from NIHR, during the conduct of the study; Dr. Metcalfe reports grants from UK National Institute of Health Research HTA programme, during the conduct of the study; Dr Barry Ingham is a consultant clinical psychologist employed by an NHS Trust which has received external funding (NIHR and charities) to support his involvement in adult autism research projects; Dr. Wiles reports grants from University of Bristol, during the conduct of the study.

Figure 1: Timeline of Events

REFERENCES

1 American Psychiatric Association Diagnostic and statistical manual of mental disorders (5th ed.) Washington, DC 2009.
2 Mannion, A, and Leader, G Co-morbidity in Autism Spectrum Disorder: A Review *Res Autism Spectr Disord* 2013; 7:1595-1616
3 Russell, AJ, Murphy, CM, Wilson, E et al. Short Report: The mental health of individuals referred for assessment of autism spectrum disorder in adulthood: A clinic report *Autism* 2016; **20(**5): 623-627
4 Whiteford HA, Degenhardt L, Rehm J, et al. Global burden of disease attributable to mental and substance use disorders: findings from the Global Burden of Disease Study 2010. *Lancet* 2013; **382**(9904): 1575-86.
5 McManus, S, Meltzer, H, Brugha, T, Bebbington, P. and Jenkins, R. (Editors) Adult Psychiatric Morbidity in England: results of a Household Survey The NHS Information Centre 2009
6 Hofvander, B, Delorme, R, Chaste, P, *et al.* Psychiatric and psychosocial problems in adults with normal-intelligence autism spectrum disorders *BMC Psychiatry* 2009; 9: 35; 1-9
7 Wigham S, Barton S, Parr JR et al. A systematic review of the rates of depression in children and adults with high functioning autism spectrum disorder *Journal of Mental Health Research in Intellectual Disabilities* 2017; **10**(4): 267-287.
8 NICE Clinical Guidelines 90 (CG90) *Depression in adults: recognition and management* National Institute for Healthcare and Excellence 2009

For peer review only - http://bmjopen.bmj.com/site/about/guidelines.xhtml

9 NICE Clinical Guidelines 142 (CG142)) *Autism Spectrum Disorder in adults: recognition and management* National Institute for Healthcare and Excellence 2012

10 Lang, R., Regester, A, Lauderdale, S et al. Treatment of anxiety in autism spectrum disorders using cognitive behaviour therapy: A systematic review *Developmental Rehabilitation* 2010; **13**(1): 53-63

11 Sukhodolsky, DG, Bloch, MH, Panza, KE et al. Cognitive behavioural therapy for anxiety in children with High-Functioning Autism: A Meta-analysis *Pediatrics* 2013; **132**(5):1341-1350

12 Spain, D, Sin, J, Chalder, T, et al. Cognitive behaviour therapy for adults with autism spectrum disorders and psychiatric co-morbidity: A Review *Res Autism Spectr Disord* 2015; 9: 151-162

13 Weston, L, Hodgekins, J, & Langdon, P E. Effectiveness of cognitive behavioural therapy with people who have autistic spectrum disorders: A systematic review and meta-analysis. *Clin Psychol Rev* 2016; 49: 41–54

14 Santomauro, D, Sheffield, J and Sofronoff, K Depression in adolescents with ASD: A pilot RCT of a group intervention *J Autism Dev Disord* 2016; **46**(2):572-588

15 McGilliveray, JA and Evert, HT Group cognitive behavioral program shows potential in reducing symptoms of depression and stress among young people with Autism Spectrum Disorders *J Autism Dev Disord* 2014; 44: 2041-51

16 Sizoo, BB and Kuiper, E. Cognitive Behaviour Therapy and Mindfulness Based Stress Reduction may be equally effective in reducing anxiety and depression in adults with Autism Spectrum Disorder *Res Dev Disabil* 2017; 64: 47-55

17 Lewis, G, Pelosi, AJ, Araya, R et al. Measuring psychiatric disorder in the community: a standardized assessment for use by lay interviewers. *Psychol Med* 1992; ***22***(2): 465-486.

18 Kroenke, K, & Spitzer, RL. The PHQ-9: a new depression diagnostic and severity measure. *Psychiatr Ann* 2002; ***32***(9):509-515

19 Cameron, I M, Crawford, JR, Lawton, K et al. Psychometric comparison of PHQ-9 and HADS for measuring depression severity in primary care *Br J Gen Pract* 2008; **58**(546): 32-36

20 Beck, A T, Steer, R A, & Brown, G K (1996) Beck Depression Inventory-II. *San Antonio*, 1996

21 Gotham, K, Unruh, K and Lord, C. Depression and its measurement in verbal adolescents and adults with autism spectrum disorder *Autism* 2015; **19**(4): 491-504

22 Williams, J B, Kobak, K A, Bech, P et al. The GRID-HAMD: standardization of the Hamilton depression rating scale. *Int Clin Psychopharmacol* 2008; ***23***(3):120-129.

23 Hamilton, M. (1960) A Rating Scale for Depression *J Neurol Neurosurg Psychiatry* 1960; 23: 56

24 Spitzer, R L, Kroenke, K, Williams, J B et al. A brief measure for assessing generalized anxiety disorder: the GAD-7 *Arch Intern Med* 2006;***166***(10):1092-1097.

25 Crawford, J R & Henry, J D The Positive and Negative Affect Schedule (PANAS): Construct validity, measurement properties and normative data in a large non-clinical sample *Br J Clin Psychol* 2004; ***43***(3): 245-265.

26 Foa, E B, Huppert, J D, Leiberg, S et al The Obsessive-Compulsive Inventory: development and validation of a short version *Psychol Assess* 2002; **14**(4): 485

27 Cadman, T, Spain, D, Johnston,P, et al Obsessive-compulsive disorder in adults with high-functioning autism spectrum disorder: what does self-report with the OCI-R tell us? *Autism Res* 2015; **8**(5): 477-85

28 Mundt, J C, Marks, I M, Shear, M K et al. The Work and Social Adjustment Scale: a simple measure of impairment in functioning *Br J Psychiatry* 2002;**180**(5):461-464

29 Herdman, M, Gudex, C, Lloyd, A et al. Development and preliminary testing of the new five-level version of EQ-5D (EQ-5D-5L). *Qual Life Res* 2011;***20***(10):1727-1736

For peer review only - http://bmjopen.bmj.com/site/about/guidelines.xhtml

30 Janssen, M F, Birnie, E, Haagsma, J A et al. (2008). Comparing the standard EQ-5D three-level system with a five-level version. *Value in Health* 2008;**11**(2):275-284
31 Ware, JE, Kosinski, M, and Keller, SD (1996) A 12-item short-form Health Survey (SF-12): Construction of scales and preliminary test of reliability *Medical Care* 1996; **32**(3): 220-233
32 Salyers, M P, Bosworth, H B, Swanson, J W et al. Reliability and validity of the SF-12 health survey among people with severe mental illness. *Medical care* 2000;**38**(11):1141-1150
33 Barrett, S L, Uljarević, M, Baker, E K et al. The Adult Repetitive Behaviours Questionnaire-2 (RBQ-2A): a self-report measure of restricted and repetitive behaviours. *J Autism Dev Disord* 2015;***45*****(**11): 3680-3692
34 Trapnell, P D, & Campbell, J D Private self-consciousness and the five-factor model of personality: distinguishing rumination from reflection. *J Pers Soc Psychol* 1999;**76**(2): 284
35 Eldridge, SM, Chan, CL. Campbell, MJ et al. CONSORT 2010 Statement: extension to randomised pilot and feasibility trials BMJ 2016; MJ,355:i5239 http://dx.doi.org/10.1136/bmj. i5239
36 Noble, M, McLennan, D, Wilkinson, K et al. The English Indices of Deprivation 2007 Communities and Local Government Publications; 2008
37 NVivo qualitative data analysis Software; QSR International Pty Ltd. Version 10, 2012
38 Braun V. & Clarke V. Using thematic analysis in psychology. *Qual Res Psychol* 2006;3:77-101
39 Boyatzis, R. *Transforming qualitative information: Thematic analysis and code development*. Thousand Oaks, CA: Sage 1998
40 Charmay K. *Constructing grounded theory: a practical guide through qualitative analysis*. London: Sage 2006

For peer review only - http://bmjopen.bmj.com/site/about/guidelines.xhtml

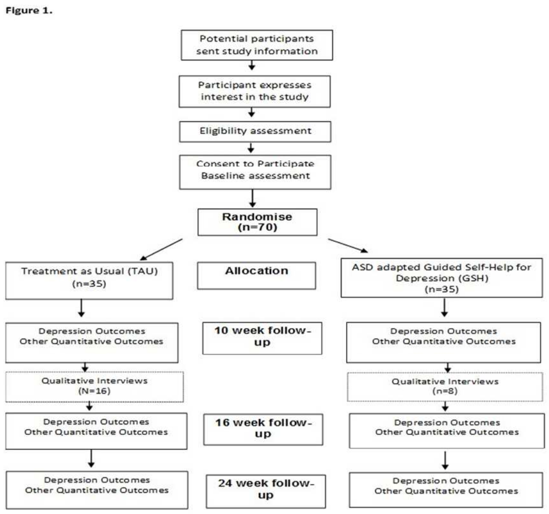

Figure 1: Timeline of Events

108x60mm (300 x 300 DPI)

review only

Table 2: CONSORT checklist of information to include when reporting a pilot trial

Section/Topic and Item No.	Standard Checklist Item	Extension for pilot trials	Page No. where item is reported
**Title and abstract**			
1a	Identification as a randomised trial in the title	Identification as a pilot or feasibility randomised trial in the title	1
1b	Structured summary of trial design, methods, results and conclusions	Structured summary of trial design, methods, results and conclusions (for specific guidance see CONSORT abstract extension for pilot trials)	2
**Introduction**			
Background and Objectives:			
2a	Scientific background and explanation of rationale	Scientific background and explanation of rationale for future definitive trial, and reasons for randomised pilot trial	3-4
2b	Specific objectives or hypotheses	Specific objectives or research questions for pilot trial	4
**Methods**			
Trial design:			
3a	Description of trial design including allocation ratio	Description of pilot trial design including allocation ratio	4
3b	Important changes to methods after trial commencement (such as eligibility criteria) with reasons	Important changes to methods after trial commencement (such as eligibility criteria) with reasons	Nil to report
Participants:			
4a	Eligibility criteria for participants		4-5
4b	Settings and locations where the data were collected		5
4c		How participants were identified and consented	5

For peer review only

Interventions:			
5	The interventions for each group with sufficient details to allow replication, including how and when they were actually administered		6-7
Outcomes:			
6a	Completely defined pre-specified primary and secondary outcome measures including how and when they were assessed	Completely defined pre-specified assessments or measurements to address each pilot trial objective specified in 2b, including how and when they were assessed	7,8,9,10
6b	Any changes to trial outcomes after the trial commenced, with reasons	Any changes to pilot trial assessments or measurements after the pilot trial commenced, with reasons	Nil to report
6c		If applicable, pre-specified criteria used to judge whether, or how, to proceed with future definitive trial	n/a
Sample size:			
7a	How sample size was determined	Rationale for numbers in the pilot trial	10
7b	When applicable, explanation of any interim analyses and stopping guidelines		n/a
Randomisation:			
Sequence generation:			
8a	Method used to generate the random allocation sequence		5
8b	Type of randomisation: details of any restriction (such as blocking and	Type of randomisation: details of any restriction (such as blocking and	5,6

For peer review only

	block size)	block size)	
Allocation concealment mechanism:			
9	Mechanism used to implement the random allocation sequence (such as sequentially numbered containers), describing any steps taken to conceal the sequence until interventions were assigned		5
Implementation:			
10	Who generated the random allocation sequence, enrolled participants and assigned participants to interventions		6
Blinding:			
11a	If done, who was blinded after assignment to interventions (e.g. participants, care providers, those assessing outcomes) and how		7
11b	If relevant, description of the similarity of the interventions		n/a
Analytical Methods:			
12a	Statistical methods used to compare groups for primary and secondary outcomes	Methods used to address each pilot trial objective whether qualitative or quantitative	10,11
12b	Methods of additional analyses, such as subgroup analyses and adjusted analyses	Not applicable	n/a
**Results**			
Participant flow (a diagram is strongly recommended)			

For peer review only

13a	For each group, the numbers of participants who were randomly assigned, received intended treatment, and were analysed for the primary outcome	For each group, the numbers of participants who were randomly assigned, received intended treatment, and were assessed for each objective	5, 6, 10
13b	For each group, losses and exclusions after randomisation, together with reasons		n/a
Recruitment:			
14a	Dates defining the periods of recruitment and follow-up		2,12
14b	Why the trial ended or was stopped	Why the pilot trial was ended or was stopped	n/a
Baseline data:			
15	A table showing baseline demographic and clinical characteristics for each group		n/a
Numbers analysed:			
16	For each group, number of participants (denominator) included in each analysis and whether the analysis was by original assigned groups	For each objective, number of participants (denominator) included in each analysis. If relevant, these numbers should be by randomised group	10
Outcomes and estimation:			
17a	For each primary and secondary outcome, results for each group, and the estimated effect size and its precision (such as 95% confidence interval)	For each objective, results including expressions of uncertainty (such as 95% confidence interval) for any estimates. If relevant, these results should be by randomised group	10
17b	For binary outcomes, presentation	Not applicable	n/a

For peer review only

	of both absolute and relative effect sizes is recommended		
Ancillary analyses:			
18	Results of any other analyses performed, including subgroup analyses and adjusted analyses, distinguishing pre-specified from exploratory	Results of any other analyses performed that could be used to inform the future definitive trial	10
Harms:			
19	All important harms or unintended effects in each group (for specific guidance see CONSORT for hamrs0		11
19a		If relevant, other important unintended consequences	n/a
**Discussion**			
Limitations:			
20	Trial limitations, addressing sources of potential bias, imprecision, and if relevant, multiplicity of analyses	Pilot trial limitations, addressing sources of potential bias and remaining uncertainty about feasibility	2,11
Generalisability:			
21	Generalisability (external validity, applicability) of the trial findings	Generalisability (applicability) of pilot trial methods and findings to future definitive trial and other studies	11
Interpretation:			
22	Interpretation consistent with results, balancing benefits and harms, and considering other relevant evidence	Interpretation consistent with pilot trial objectives and findings, balancing potential benefits and harms, and considering other	n/a at protocol stage

For peer review only

		relevant evidence	
22a		Implications for progression from pilot trial to future definitive trial, including any proposed amendments	n/a
**Other Information**			
Registration:			
23	Registration number and name of trial registry	Registration number of pilot trial and name of trial registry	2
Protocol:			
24	Where the full trial protocol can be accessed, if available	Where the pilot trial protocol can be accessed, if available	12
Funding:			
25	Sources of funding and other support (such as supply of drugs), role of funders		1
26		Ethical approval or approval by research review committee, confirmed with reference number	2

For peer review only
