# Supplementary material for: Protocol for a feasibility study and randomised pilot trial of a low-intensity psychological intervention for depression in adults with autism: the Autism Depression Trial (ADEPT)
Source: BMJ Open. 2017 Dec 3;7(12):e019545. doi: 10.1136/bmjopen-2017-019545 (PMC5736092; doi:10.1136/bmjopen-2017-019545)
Supplement: Reviewer comments [file bmjopen-2017-019545.reviewer_comments.pdf]

PEER REVIEW HISTORY

BMJ Open publishes all reviews undertaken for accepted manuscripts. Reviewers are asked to complete a checklist review form (**http://bmjopen.bmj.com/site/about/resources/checklist.pdf**) and are provided with free text boxes to elaborate on their assessment. These free text comments are reproduced below.

ARTICLE DETAILS

**TITLE (PROVISIONAL)**	Protocol for a feasibility study and randomised pilot trial of a low intensity psychological intervention for depression in adults with autism: The Autism Depression Trial (ADEPT)
**AUTHORS**	Russell, Ailsa; Cooper, Kate; Barton, stephen; Ensum, Ian; Gaunt, Daisy; Horwood, Jeremy; Ingham, Barry; Kessler, David; Metcalfe, Chris; Parr, Jeremy; Rai, Dheeraj; Wiles, Nicola

This article was not externally reviewed at BMJ Open. Protocols that have been independently assessed prior to submission to BMJ Open are usually fast-tracked to publication on the grounds that further substantial changes will not be possible. This independent assessment will usually be external, independent review for both a substantial grant award from a non-commercial or government funder and ethics approval.
